# Efficacy, safety, and characteristics of adoptive tumor-infiltrating lymphocyte therapy in solid tumours: a systematic review and meta-analysis

**DOI:** 10.3389/fonc.2026.1859306

**Published:** 2026-07-22

**Authors:** Haripriya Parapparambil Surendran, Gopal Gopisetty, Senthil Jagannathan Rajappa, T. Subramanyeshwar Rao, M. K. Unnikrishnan

**Affiliations:** 1 Indo-American Cancer Research Foundation, Basavatarakam Indo-American Cancer Hospital and Research Institute, Hyderabad, Telangana, India; 2 Basavatarakam Indo-American Cancer Hospital and Research Institute, Hyderabad, Telangana, India; 3 Amrita School of Pharmacy, Amrita Vishwa Vidyapeetham, Ernakulam, Kerala, India

**Keywords:** adoptive cell therapy, meta-analysis, solid tumors, systematic review, tumor-infiltrating lymphocytes

## Abstract

**Purpose:**

Adoptive tumor-infiltrating lymphocyte (TIL) therapy is an established personalized cellular immunotherapy with demonstrated activity in selected solid tumors, particularly metastatic melanoma. However, clinical outcomes, safety, and manufacturing feasibility vary across tumor types and treatment strategies. This systematic review evaluates the efficacy, safety, and operational characteristics of TIL therapy across solid malignancies.

**Methods:**

This systematic review was conducted in accordance with PRISMA guidelines. PubMed, Scopus, the Cochrane Central Register of Controlled Trials, and the WHO International Clinical Trials Registry Platform were searched from inception to 31 December 2025. Eligible studies included clinical trials and observational studies evaluating autologous TIL therapy in solid tumors. Due to substantial clinical and methodological heterogeneity, quantitative synthesis was restricted to clinically comparable cohorts, predominantly melanoma studies reporting objective response rates (ORR). A single-arm random-effects meta-analysis using the Freeman-Tukey transformation was performed. All other outcomes, including survival, safety, manufacturing success, and resection-to-infusion time, were synthesized narratively.

**Results:**

Thirty-eight studies were included: 5 randomized controlled trials (RCTs), 22 prospective non-randomized studies, and 11 retrospective analyses, spanning melanoma and 8 other solid tumour types. Meta-analysis of 18 melanoma single-arm cohorts demonstrated a pooled objective response rate (ORR) of 42% (95% CI 37%–47%; I² = 33.1%; prediction interval 29%–56%) under a random-effects model. In the phase III RCT (Rohaan et al.), TIL therapy produced superior ORR (49% vs. 21%) and progression-free survival (median 7.2 vs. 3.1 months; HR 0.50, 95% CI 0.35–0.72) compared with ipilimumab. Subgroup analysis by TIL product type revealed a statistically significant difference (χ² = 7.25, p = 0.0266): tumor-reactive TIL products pre-screened ex vivo for antigen-specific reactivity showed the highest pooled ORR at 50% (95% CI 35%–64%), followed by young TIL at 43% (95% CI 29%–58%) and bulk TIL at 36% (95% CI 31%–42%); this observation is based on only 4 cohorts with a limited aggregate patient number and should be regarded as hypothesis-generating. No significant difference in ORR was observed by lymphodepletion status (p = 0.9544). Evidence in non-melanoma solid tumours was limited and heterogeneous, with generally lower response rates. Safety profiles were consistent across studies and primarily attributable to lymphodepleting chemotherapy and interleukin-2 administration, including haematologic and cytokine-related toxicities; treatment-related mortality was uncommon. Manufacturing success rates were high across contemporary cohorts, with a resection-to-infusion time typically spanning 4–6 weeks.

**Conclusion:**

TIL therapy demonstrates consistent and clinically meaningful antitumor activity in melanoma, while evidence in non-melanoma tumors remains limited and heterogeneous. Future studies should prioritize biomarker-driven patient selection, optimization of manufacturing and conditioning strategies, and rational combination approaches to expand its applicability.

**Systematic review registration:**

https://www.crd.york.ac.uk/PROSPERO/, identifier CRD420261291389.

## 1 Introduction

Adoptive cellular immunotherapy has emerged as a transformative strategy in oncology, with tumor-infiltrating lymphocyte (TIL) therapy representing one of the earliest and most clinically validated forms of personalized cell therapy. Unlike genetically engineered approaches, TIL therapy harnesses the endogenous polyclonal antitumor T-cell repertoire, extending directly from the tumor microenvironment, which preserves native tumor specificity and minimizes the need for predefined antigen targets (1). Early clinical studies, particularly in metastatic melanoma, demonstrated robust objective responses in heavily pre-treated patients including those refractory to cytokine therapy (high-dose IL-2, interferon-α), chemotherapy (dacarbazine), and subsequently CTLA-4 blockade, establishing TIL therapy as a promising modality for refractory solid tumors (2, 3).

Treatment outcomes appear highly dependent on multiple interrelated factors, including lymphodepletion intensity, interleukin-2 (IL-2) regimens (high vs low dose), TIL manufacturing protocols, and product enrichment strategies (4). Furthermore, contemporary trials incorporate many modifications such as CD8^+^ enrichment, dendritic cell co-administration, and total body irradiation (TBI) conditioning, adding substantial heterogeneity across studies (4–6). Heterogeneity complicates cross-trial comparisons and creates uncertainty regarding the relative contribution of specific preparative strategies to clinical benefit.

In parallel, the expanding clinical pipeline of TIL therapy has highlighted important practical considerations beyond efficacy. Safety profiles are often dominated by lymphodepletion and IL-2-related toxicities, while real-world implementation depends critically on manufacturing feasibility, product success rates, and resection-to-infusion time. Although several systematic reviews have summarized TIL outcomes in selected tumor types, comprehensive evidence that integrates efficacy, safety, and operational feasibility, across solid tumors, remains limited. In particular, the magnitude of activity of pooled treatment modalities and the consistency of manufacturing performance across heterogeneous clinical settings have not been fully characterized (7, 8).

To address these gaps, we conducted a systematic review of clinical studies evaluating adoptive TIL therapy in solid tumors. Our objectives were to (i) synthesize qualitative evidence of clinical efficacy across differing TIL preparative strategies, (ii) estimate the pooled objective response rate using single-arm meta-analysis of TIL-treated cohorts in melanoma, and (iii) comprehensively evaluate safety outcomes, manufacturing success, and resection-to-infusion time to inform the translational readiness of TIL therapy in contemporary oncology practice.

## 2 Methods

### 2.1 Study design and reporting

This systematic review was conducted in accordance with the Preferred Reporting Items for Systematic Reviews and Meta-Analyses (PRISMA) 2020 guidelines and adhered to established methodological standards for systematic reviews. The methodological approach was designed to integrate evidence across heterogeneous study designs, including randomized controlled trials, non-randomized comparative studies, single-arm interventional trials, and retrospective analyses, while applying appropriate strategies for qualitative and quantitative synthesis. (Prospero registration number: CRD420261291389) (9).

### 2.2 Data sources and search strategy

We conducted a comprehensive literature search across PubMed, Scopus, the Cochrane Central Register of Controlled Trials (CENTRAL), and the World Health Organization International Clinical Trials Registry Platform (WHO ICTRP) from database inception to 31 December 2025. The search strategy combined controlled vocabulary and free-text terms related to “tumor-infiltrating lymphocytes, “ “adoptive cell therapy, “ and “solid tumors.” Reference lists of eligible studies and relevant reviews were manually screened to identify additional records. We included only those studies published in English.

### 2.3 Eligibility criteria

Studies were included if they met the following criteria: (1) clinical trials (randomized or non-randomized) or observational studies evaluating autologous TIL therapy in solid tumors; (2) reported extractable data on at least one clinical outcome (e.g., objective response rate, survival outcomes, or safety); and (3) provided sufficient methodological detail regarding TIL manufacturing or treatment protocols.

We excluded non-human studies, reviews, meta-analyses, editorials, conference abstracts without full data, case reports, and studies lacking accessible full text. Studies with overlapping patient populations were carefully assessed, and avoided duplication by considering the most comprehensive dataset.

### 2.4 Study selection

All retrieved records were imported into reference management software, and duplicates were removed. Titles and abstracts were screened, followed by full-text assessment of potentially eligible studies. Disagreements were resolved through discussion with senior reviewers until consensus was reached.

### 2.5 Data extraction

Data were extracted using a standardized and pilot-tested template. Extracted variables included study characteristics (author, year, country, design, and phase), patient population (tumor type, disease stage, prior therapies), intervention details (TIL source, selection strategy, lymphodepletion, IL-2 regimen, and cell dose), and outcomes. Clinical efficacy outcomes included objective response rate (ORR), complete response (CR), partial response (PR), progression-free survival (PFS), and overall survival (OS). For PFS and OS, the start timepoint was extracted as reported in each individual study and noted in the data extraction form; this varied across trials (from randomization, from TIL infusion, or from study inclusion), and this variability was accounted for narratively in the synthesis and in the risk-of-bias assessment. Safety outcomes included grade ≥3 adverse events and treatment-related mortality. We also gathered operational parameters such as manufacturing success rate and resection-to-infusion time, when available. Data extraction was cross-verified by senior investigators, and discrepancies were resolved by consensus.

### 2.6 Data synthesis

All included studies were initially summarized using qualitative synthesis, stratified by study design and tumor type. Given substantial clinical and methodological heterogeneity across tumor types, treatment protocols, and outcome reporting, quantitative synthesis was restricted to clinically homogeneous subsets. Specifically, single-arm meta-analysis was performed predominantly for melanoma cohorts reporting objective response rates, where sufficient consistency in treatment paradigms and outcome definitions was observed. Comparative studies and non-melanoma cohorts were synthesized narratively.

### 2.7 Risk of bias assessment

Risk of bias was assessed based on study design. Randomized controlled trials were evaluated using the Cochrane Risk of Bias 2 (RoB 2) tool (10), while non-randomized comparative studies were assessed using the ROBINS-I tool (11). Single-arm interventional studies, which constituted the majority of included evidence, and also formed the basis of the quantitative synthesis, were not formally assessed using ROBINS-I, because this tool is designed for comparative studies. Instead, methodological quality of single-arm studies was evaluated descriptively across five pre-specified domains: (1) patient selection and eligibility criteria reporting; (2) outcome assessment method and use of standardized response criteria; (3) follow-up adequacy and completeness; (4) reporting completeness including ITT vs. per-protocol analysis; and (5) manufacturing and feasibility reporting. These domains are consistent with components of the Newcastle-Ottawa Scale adapted for single-arm studies and the MINORS checklist. Retrospective studies were also assessed descriptively, with particular attention to selection bias and confounding. Risk-of-bias assessments were used to contextualize the overall quality of evidence but were not incorporated into quantitative weighting, given that the primary meta-analysis was based on single-arm cohorts.

### 2.8 Statistical analysis

The primary endpoint was the ORR. For melanoma single-arm studies, pooled proportions were estimated using a random-effects meta-analysis of proportions. To account for instability in proportions, event rates were transformed using the Freeman-Tukey double arcsine transformation, and pooled estimates with 95% confidence intervals (CIs) were calculated. Back-transformed pooled estimates are presented. Between-study heterogeneity was assessed using the Cochran Q test and quantified with the I² statistic, with thresholds of 25%, 50%, and 75% representing low, moderate, and high heterogeneity, respectively. Additionally, 95% prediction intervals were calculated to reflect the expected range of true effects in future studies. Pre-specified subgroup analyses were performed to explore potential sources of heterogeneity, including TIL product type (bulk TIL, young TIL, and tumor reactive TIL) and use of lymphodepletion. Subgroup differences were evaluated using the χ² test. Publication bias was assessed by visual inspection of funnel plots. Given the limited number of studies in certain analyses, formal statistical tests for funnel plot asymmetry were not performed. Sensitivity analyses were conducted using a leave-one-out approach, in which the pooled estimate was recalculated after sequential exclusion of each study to assess the influence of individual studies on the overall effect size.

Because of substantial clinical and methodological heterogeneity, outcomes of randomized controlled trials and non-randomized comparative studies were not pooled quantitatively, but descriptively. Similarly, studies involving non-melanoma tumor types were not included in meta-analysis and were analyzed qualitatively. All statistical analyses were performed using R software. Meta-analyses were conducted using the meta package (metaprop function), applying a random-effects model with the DerSimonian-Laird estimator.

## 3 Results

### 3.1 Study selection

**Figure 1:**
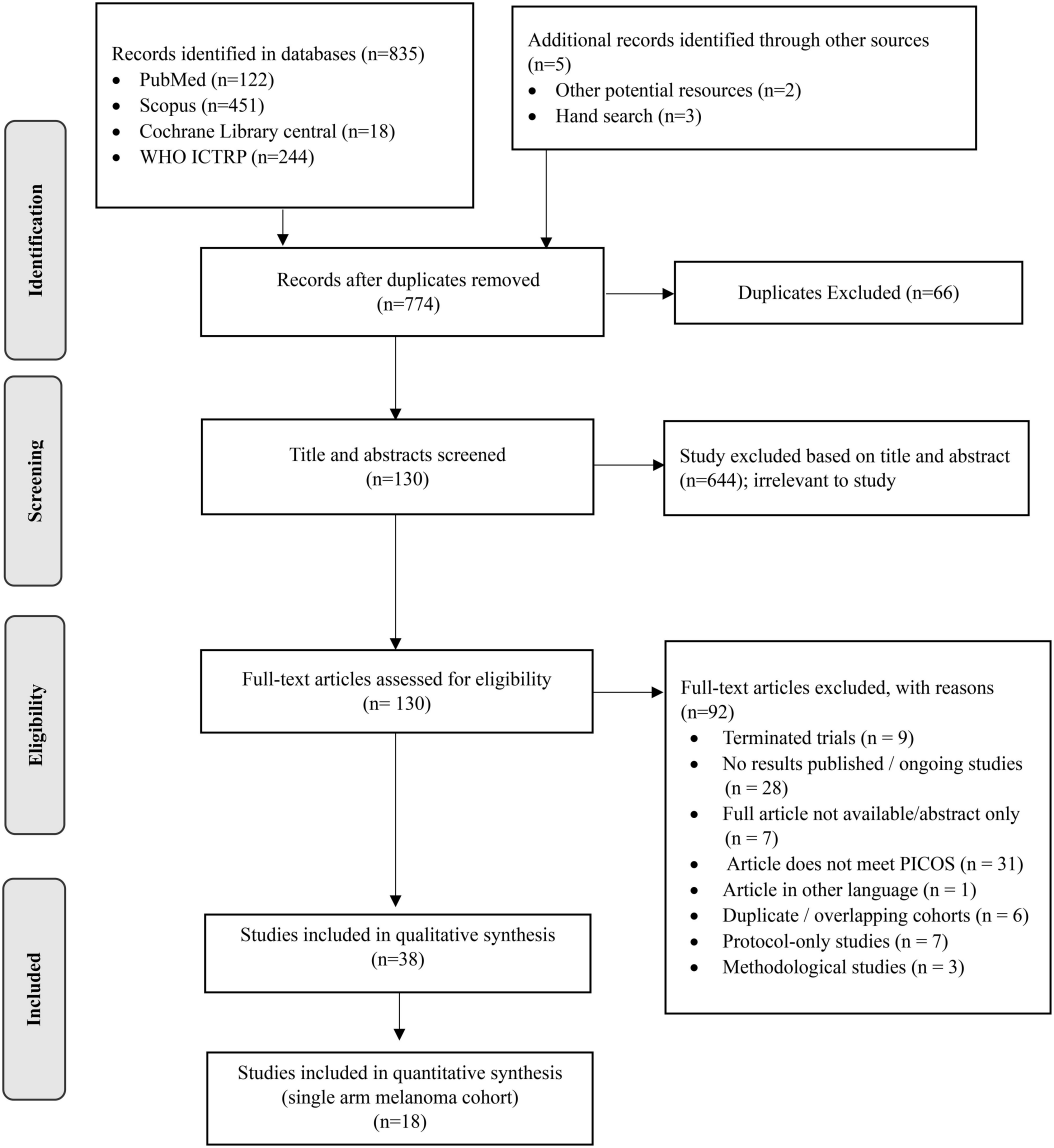
Flow diagram of study selection process (PRISMA flowchart).

The systematic search identified 840 records, including 835 from electronic databases and 5 from other sources (3 via manual hand-search of reference lists and 2 from grey-literature sources or clinical trial registries not captured by the primary database search). After removing 66 duplicates, 774 records were screened by title and abstract, of which 644 were excluded. Out of 130 full-text articles assessed for eligibility, 92 studies were excluded due to predefined reasons, including absence of results, non-eligibility according to PICOS criteria, unavailable full texts, and duplicate cohorts. Finally, 38 studies were included in the qualitative synthesis, comprising 27 melanoma studies and 11 studies in other solid tumour types. Of these, 18 melanoma single-arm studies were included in the quantitative synthesis for pooled analysis of ORR (**Figure 1**). The detailed study selection process is illustrated in **Figure 1** (PRISMA flow diagram).

### 3.2 Study characteristics

A total of 38 studies evaluating TIL-based adoptive cell therapy across solid tumors were included. Melanoma accounted for the largest body of evidence, comprising RCTs, prospective single-arm studies, and retrospective cohort analyses. Outside melanoma, evidence was limited and largely non-randomized, with studies in ovarian cancer, colorectal cancer, NSCLC, nasopharyngeal carcinoma, head and neck squamous cell carcinoma, hepatocellular carcinoma, urothelial carcinoma, and osteosarcoma.

The melanoma evidence base was methodologically heterogeneous. Early adjuvant melanoma trials evaluated TIL plus IL-2 without lymphodepletion later metastatic melanoma studies predominantly used non-myeloablative cyclophosphamide/fludarabine lymphodepletion, followed by TIL infusion and either high-dose bolus IL-2 (720, 000 IU/kg IV q8h to tolerance), attenuated high-dose IL-2 (600, 000 IU/kg IV, capped at 6 doses, as in the lifileucel trials), low-dose subcutaneous IL-2, or decrescendo continuous infusion regimens (**Supplementary Table 1**). TIL products also varied across studies and included three main manufacturing categories: (1) bulk TIL polyclonal lymphocytes expanded from tumor fragments without antigen-specific pre-selection, representing the most commonly used approach; (2) young TIL-rapidly expanded, minimally manipulated products using short-term culture protocols (typically 2–3 weeks), used in Besser et al. (Israel), Andersen et al. (Denmark), van den Berg et al., Sarnaik et al. (lifileucel), and Chesney et al.; and (3) tumor-reactive TIL-products pre-screened ex vivo for antigen-specific reactivity using co-culture IFN-γ release assays, used in Goedegebuure et al., Rosenberg et al. (2011), Chandran et al., and Pilon-Thomas et al. Several studies described products with features of more than one category (e.g., bulk expansion followed by functional testing); these were classified according to their predominant manufacturing strategy. Most non-randomized melanoma cohorts enrolled heavily pretreated patients, frequently after prior cytokine therapy, CTLA-4 blockade, PD-1/PD-L1 inhibition, or targeted therapy. In contrast, non-melanoma studies were generally small, exploratory, and feasibility-oriented (**Supplementary Table 1**).

### 3.3 Risk of bias

**Figure 2:**
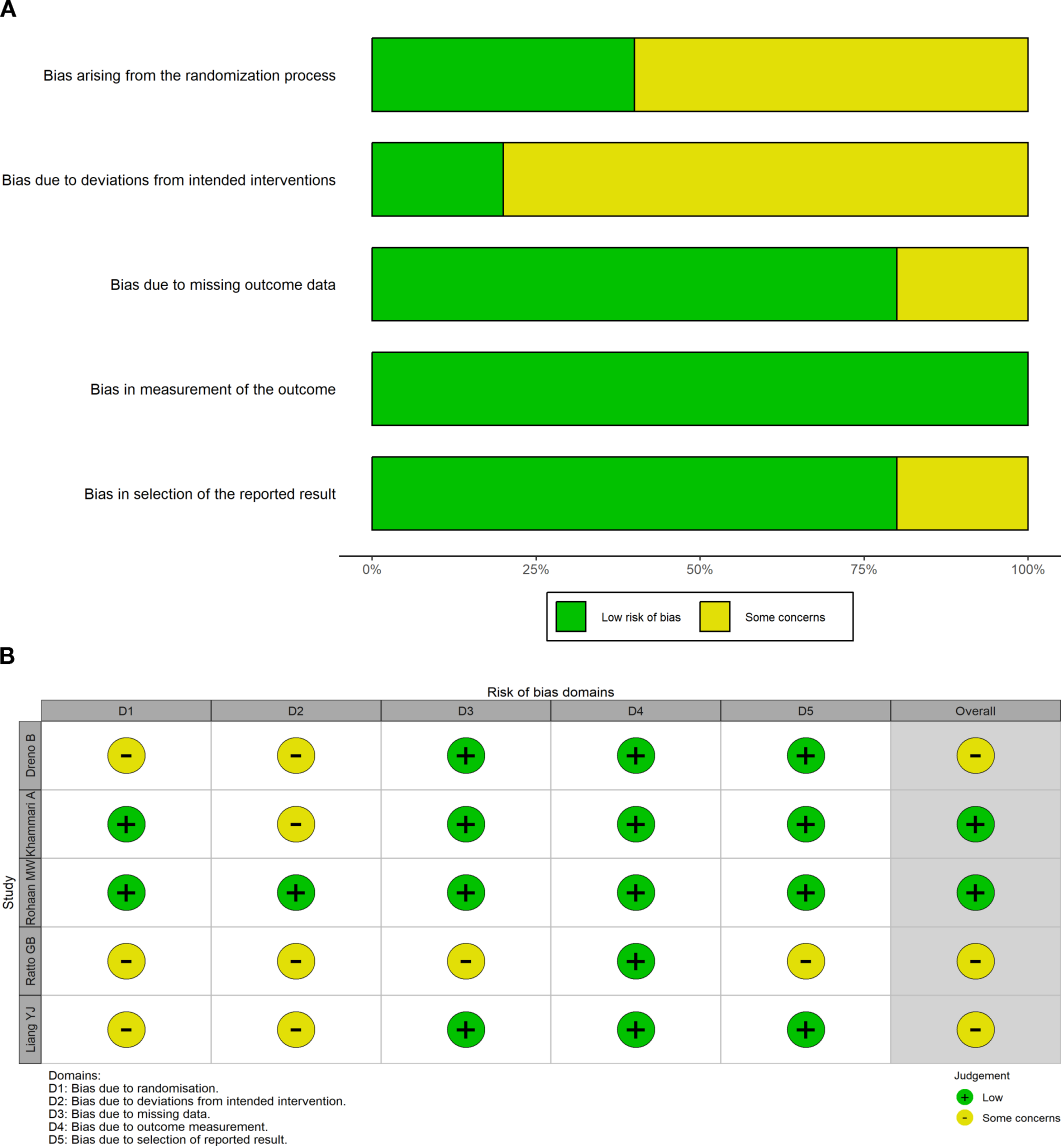
**(a)** Risk of Bias Graph presented across all RCTs. **(b)** Risk of Bias Summary presented across all RCTs.

**Figures 2a, b** depict risk of bias for RCTs, assessed by Cochrane Risk of Bias 2 (RoB 2) tool. Bias arising from the randomization process was low in 3/5 studies (60%), but higher in the remaining studies. Four out of five studies (80%) were biased because of deviations from intended interventions had the highest proportion of concerns, with classified as having some concerns. Bias due to missing outcome data was low in 4/5 studies (80%), with concerns in only one study. All studies (100%) were judged as low risk in outcome measurement. Bias in selection of the reported result was low in 4/5 studies (80%), with only one study having some concerns. Overall, two studies (Khammari et al (13). and Rohaan et al. (14)) had a low risk of bias, while the remaining three studies (Dréno et al. (12), Ratto et al. (41), and Liang et al. (42)) had some concerns, with no study assessed as high risk.

**Figure 3:**
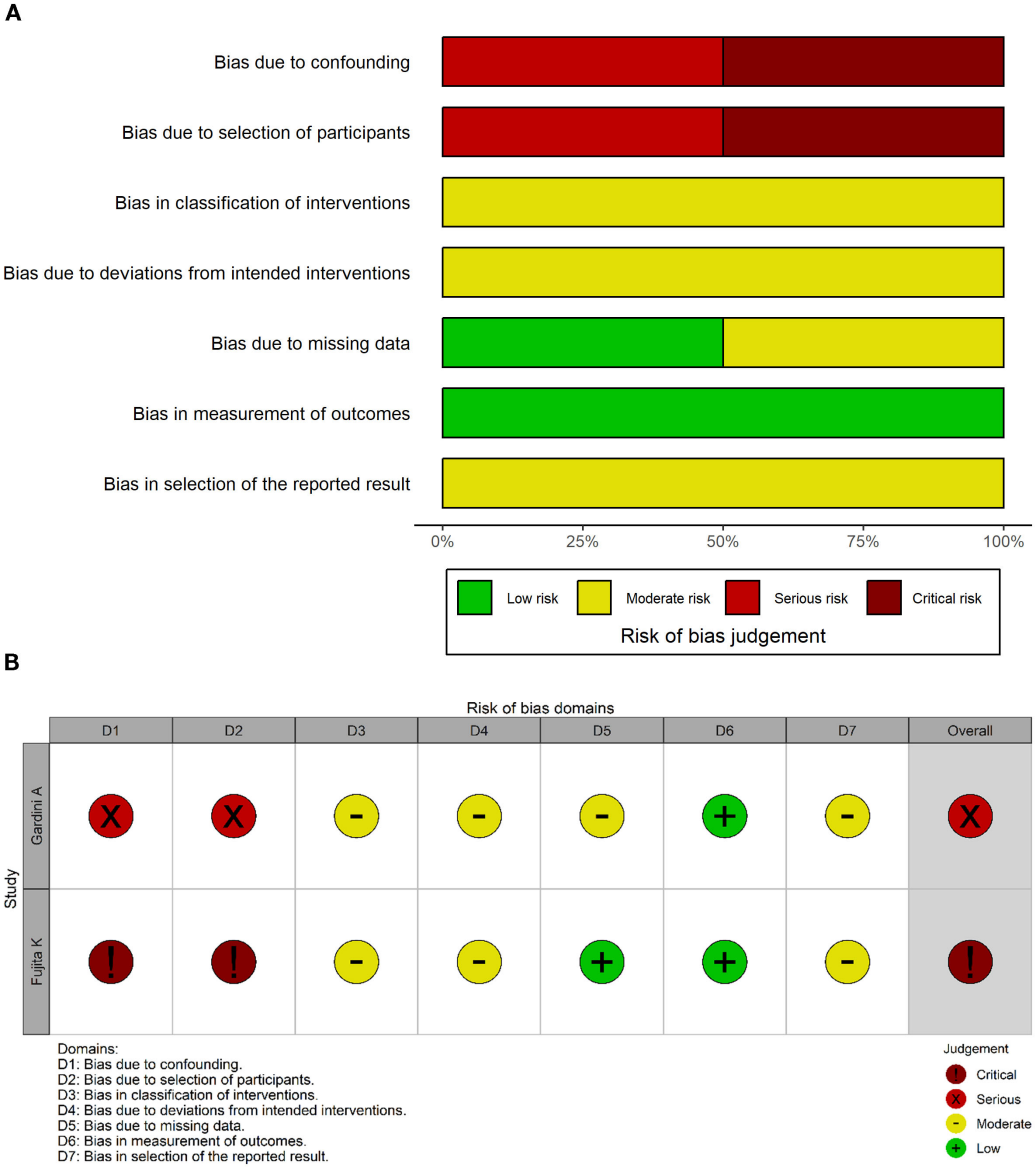
**(a)** Risk of Bias graph for Non-Randomized Controlled Studies. **(b)** Risk of Bias Summary for Non-Randomized Controlled Studies.

**Figures 3a, b** depict risk of bias for non-randomized studies, assessed by the ROBINS-I tool. Across all included studies, bias from confounding and participant selection was consistently judged as serious to critical risk. Bias related to classification of interventions and deviations from intended interventions was uniformly assessed as moderate. Bias due to missing data varied across studies, with approximately half of the studies classified as low risk. All studies were judged to be at low risk of bias in outcome measurement. Bias in the selection of the reported result was consistently assessed as moderate risk (38, 39).

Formal risk of bias tools were not applied to single-arm studies. Instead, methodological quality was evaluated descriptively. Across these studies, patient populations were predominantly heavily pretreated, with variability in the reporting of eligibility criteria. Outcome assessment was performed using standardized response criteria in most studies; however, reporting of assessor blinding was limited. Follow-up duration varied across studies, and reporting completeness was inconsistent, particularly for time-to-event outcomes (**Supplementary Table 2**). Retrospective studies were assessed descriptively, with emphasis on methodological limitations. All studies were subject to selection bias due to non-random inclusion of patients, and confounding arising from differences in baseline characteristics and prior treatments. Outcome assessment was based on retrospective data collection, with variability in reporting of response criteria and follow-up duration. Reporting of outcomes was incomplete in several studies, particularly for survival endpoints (**Supplementary Table 3**).

### 3.4 Primary outcome

#### 3.4.1 Melanoma/metastatic melanoma

Three randomized trials were included (12–14). In the adjuvant setting, Dréno et al. reported a median overall survival of 29.5 months in the TIL + IL-2 arm versus 20.0 months in the IL-2-alone arm (12). Khammari et al. reported a 5-year overall survival of 70.8% in the TIL + IL-2 group compared with 52.2% in the control group (HR 0.503, 95% CI 0.195–1.30) (13). In advanced melanoma, Rohaan et al. reported an objective response rate of 49% (41/84) in the TIL arm compared with 21% (18/84) in the ipilimumab arm. Complete response rates were 20% vs 7%, and partial response rates were 29% vs 14%, respectively. Median progression-free survival was 7.2 months versus 3.1 months (HR 0.50, 95% CI 0.35–0.72). Median overall survival was 25.8 months in the TIL arm and 18.9 months in the control arm (HR 0.83, 95% CI 0.54–1.27) (14).

No prospective non-randomized dual-arm melanoma trials were identified in the main prospective evidence base; however, retrospective comparative cohort analyses provided some non-randomized comparative data. Mehta et al. examined outcomes in patients with M1c melanoma according to brain metastasis status and found that patients without brain metastases had higher systemic ORR (49%) than those with treated (33%) or untreated brain metastases (33%); intracranial ORR in untreated brain metastases was 28%. Both progression-free and overall survival were significantly worse in patients with untreated brain metastases (36). McClelland et al. evaluated ACT-TIL in acral melanoma using a non-acral cohort as an internal comparator and reported an ORR of 43%, median PFS of 3.5 months, and median OS of 13 months, indicating that activity was retained even in this biologically distinct low-TMB melanoma subtype (37). Saint-Jean et al. reported more modest results in an elderly, heavily pretreated cohort, with an ORR of 20% and disease control rate of 40% (35).

The prospective single-arm melanoma studies showed substantial heterogeneity in design, eligibility, manufacturing approach, and treatment regimen, but overall demonstrated consistent antitumor activity (15, 17–22, 24–28, 30–34, 44). Reported ORRs ranged from 0% in the earliest pilot study by Baars et al (15). to 75% in the BRAF-mutated cohort pretreated with vemurafenib (EUCTR_2014-001419-38). Several studies reported ORRs in the 30%-56% range, including Dudley et al. (51%) (17), Rosenberg et al. (56%) (18), Radvanyi et al. (48.4%) (19), Andersen et al. (42%) (25), Forget et al. (42%) (27), Sarnaik et al. (36%) (33), and Chesney et al. (31.4%) (33). Complete responses were observed repeatedly across studies, and in some cohorts these responses were notably durable. Rosenberg et al. reported ongoing complete responses lasting 37-82+ months (18, 30), van den Berg et al. documented complete responses persisting beyond 7–9 years (30), and several other studies reported ongoing complete responses beyond 1–3 years (**Supplementary Table 4**). Of particular note for durability, the 5-year follow-up data of lifileucel (Medina et al., 2025) demonstrated sustained long-term responses in a subset of melanoma patients who achieved complete or durable partial responses in the pivotal C-144–01 cohort, further supporting the potential for prolonged remission with TIL therapy in this indication (23).

##### 3.4.1.1 Single-arm meta-analysis of melanoma studies

Quantitative synthesis was performed for 18 melanoma single-arm cohorts (15–17, 19–22, 24–33, 40). The pooled analysis demonstrated a summary ORR of 42% under the random-effects model (95% CI 37%–47%), with a prediction interval of 29%–56%. Statistical heterogeneity was moderate (I² = 33.1%), indicating that although study-level variability was present, the overall treatment effect was relatively consistent across cohorts (**Figure 4.1**).

**Figure 4:**
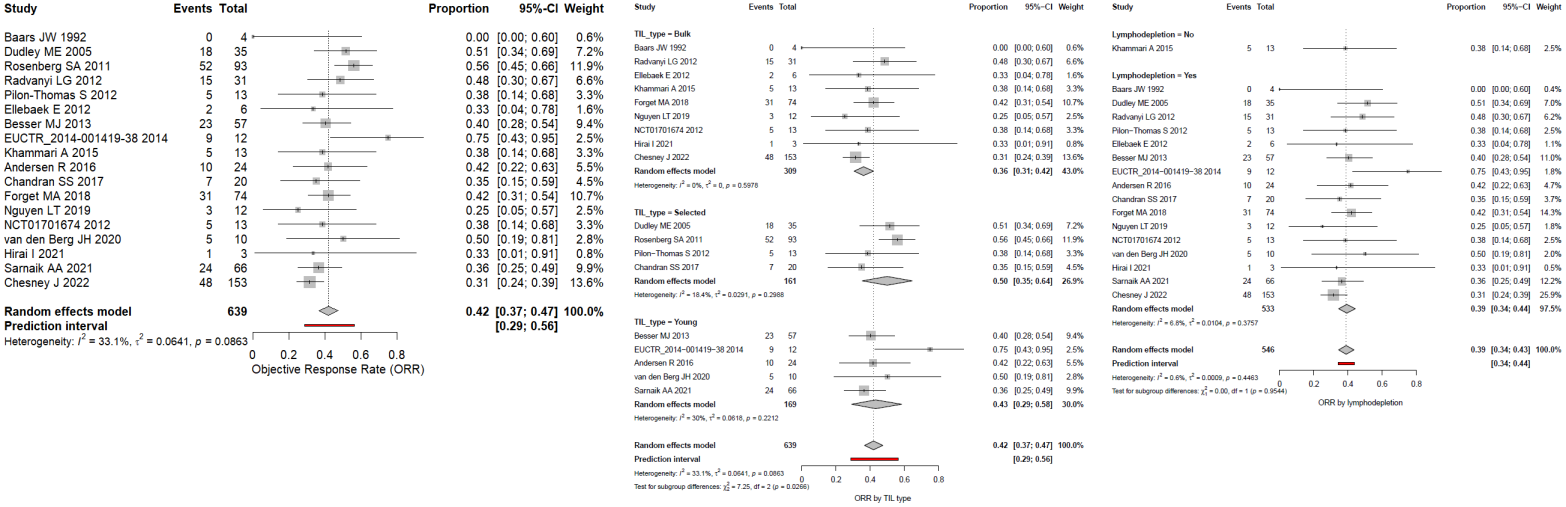
(1) Pooled ORR of TILs in melanoma single-arm cohort trials. (2) Pooled ORR by TILs manufacturing type in melanoma single-arm cohort trials. (3) Pooled ORR by lymphodepletion in melanoma single-arm cohort trials.

###### 3.4.1.1.1 Subgroup analysis

Subgroup analysis according to TIL product type (bulk TILs: Unselected tumor-derived lymphocytes; young TILs: Short-term expanded, minimally manipulated TILs; ‘tumor-reactive TIL: TILs enriched for CD8^+^ cytotoxic T cells): TILs pre-screened ex vivo for antigen-specific reactivity using co-culture assays; no study in the included evidence base used CD8^+^ immunomagnetic enrichment as its primary selection strategy) demonstrated a statistically significant difference in ORR (test for subgroup differences: χ² = 7.25, df = 2, p = 0.0266). Studies using tumor-reactive TIL products (n=4 cohorts, limited aggregate patient number) showed the highest pooled ORR at 50% (95% CI 35%–64%); this observation should be regarded as hypothesis-generating only, as it is based on a small number of cohorts. Young TIL showed 43% (95% CI 29%–58%) and bulk TIL showed 36% (95% CI 31%–42%) (**Figure 4.2**). In contrast, subgrouping by lymphodepletion status did not show a significant difference in pooled ORR (p = 0.9544). The pooled ORR was 38% (95% CI 14%–68%) in the small subgroup without lymphodepletion and 39% (95% CI 34%–44%) in studies using lymphodepletion (**Figure 4.3**).

###### 3.4.1.1.2 Publication bias

**Figure 5:**
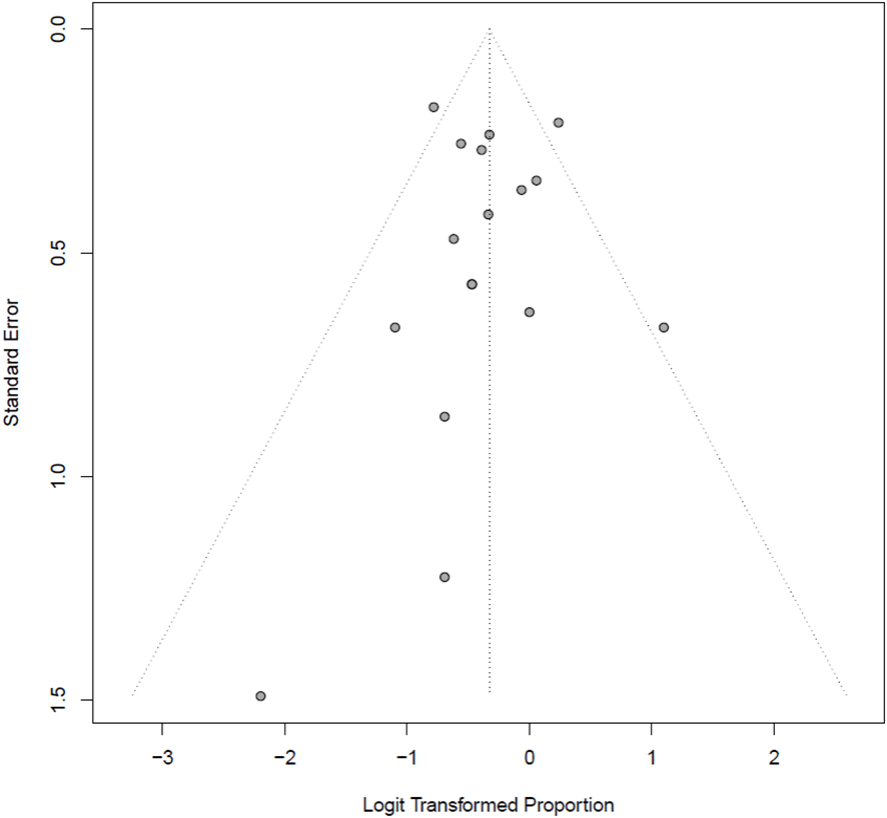
Funnel plot for assessment of publication bias in melanoma single-arm studies.

Visual inspection of the funnel plot did not demonstrate clear asymmetry, although dispersion was observed among smaller studies at higher standard errors (**Figure 5**).

###### 3.4.1.1.3 Sensitivity analysis

**Figure 6:**
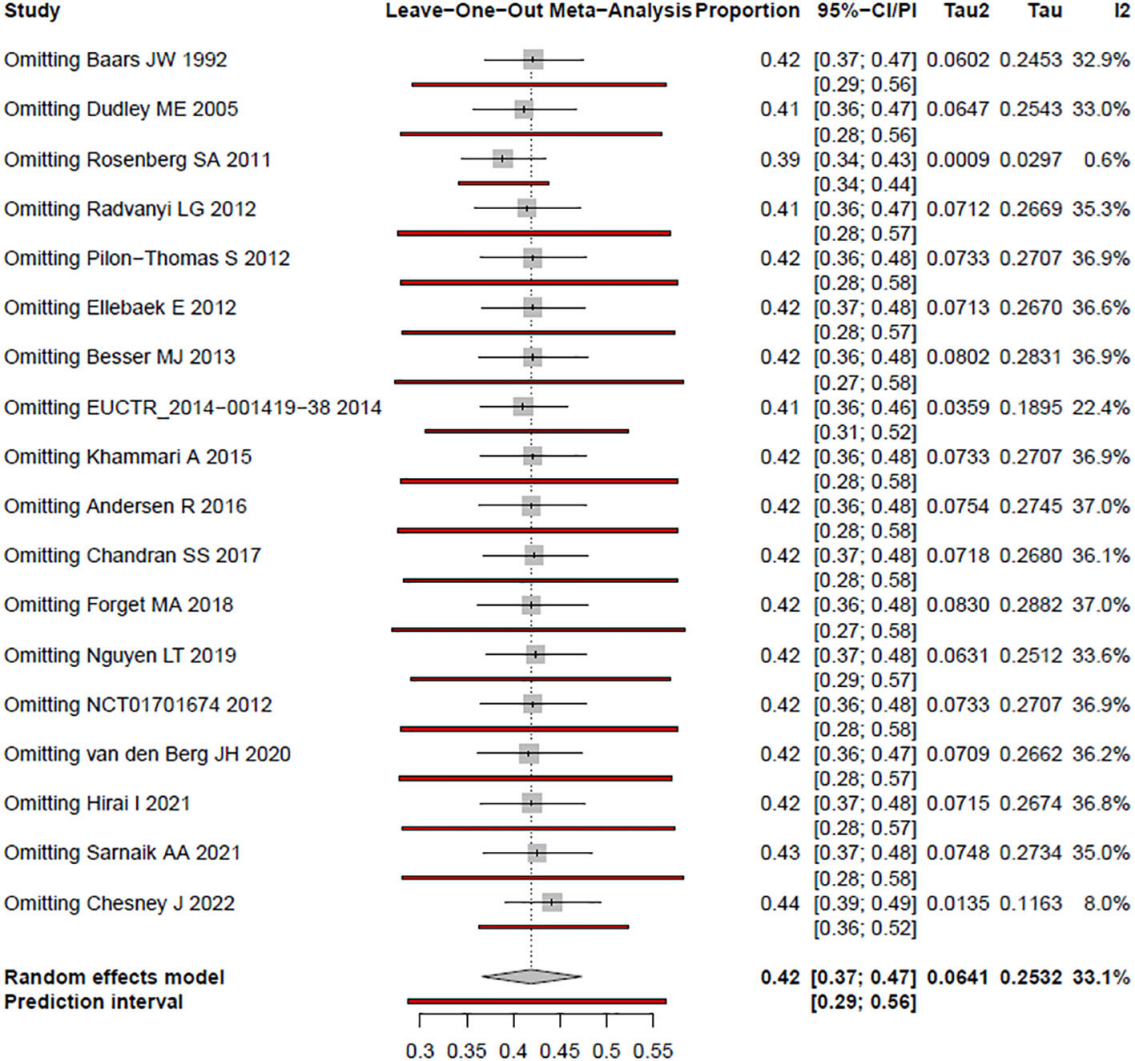
Leave-one-out sensitivity analysis of pooled ORR in melanoma single-arm studies.

Leave-one-out sensitivity analysis showed pooled ORR estimates ranging from 39% to 44% following sequential exclusion of individual studies. The lowest estimate was observed after exclusion of Rosenberg et al. (39%, 95% CI 34%-43%), and the highest after exclusion of Chesney et al. (44%, 95% CI 39%-49%) (**Figure 6**).

#### 3.4.2 Other cancers- primary outcome

Evidence outside melanoma was sparse and largely exploratory. In ovarian cancer, Fujita et al. reported improved 3-year disease-free survival with adjuvant TIL therapy after chemotherapy (82.1% vs 54.5%, p < 0.05) in a non-randomized controlled study (38). By contrast, recurrent ovarian cancer single-arm studies were smaller and focused mainly on feasibility; Kverneland et al. reported 1 partial response among 6 patients (16.7%) with a 100% disease control rate, median PFS of about 2.8 months, and median OS of about 7 months (43).

In colorectal cancer, Gardini et al. evaluated adjuvant TIL therapy after radical resection of liver metastases and found no clear overall survival advantage, with 5-year OS rates of 25% in the TIL group and 38% in controls (p = 0.7) (39). In a modern phase II multi-cohort study of refractory solid tumors, the colorectal/pancreatic/ovarian cohorts treated with TIL therapy showed 0% ORR, although disease control was observed in 62.5% of patients, with median PFS 2.53 months and median OS 18.86 months (46).

In NSCLC, the single identified randomized study suggested a survival signal favoring TIL plus IL-2 after resection, with median OS reported as 22.4 months in the adoptive immunotherapy arm versus 14.1 months in controls (41).

In nasopharyngeal carcinoma, Liang et al. reported very high response rates in both arms, with ORR 97.4% for CCRT + TIL and 94.9% for CCRT alone. However, hazard ratios for progression-free survival (HR 1.08, 95% CI 0.62–1.89) and overall survival (HR 1.15, 95% CI 0.57–2.29) did not indicate a clear survival benefit from adding TIL therapy (42).

In head and neck squamous cell carcinoma, activity varied by manufacturing cohort in the phase II LN-145/LN-145-S1 study. ORR ranged from 37.5% in cohort 1 to 5.9% and 0% in later cohorts, while disease control rates remained relatively high (70.6%–87.5%), suggesting biologic activity despite modest objective responses in some cohorts (45).

In hepatocellular carcinoma, Jiang et al. primarily assessed safety in the post-surgical setting; however, 12 of 15 patients (80%) were reported as having no evidence of disease during follow-up, with recurrence documented in 3 patients (44). In osteosarcoma, Wang et al. observed no objective responses, but disease stabilization in 81.8% of patients, with median PFS 4.1 months and median OS 7.6 months (47). Evidence in urothelial carcinoma was feasibility-focused and insufficient to draw firm conclusions regarding efficacy (48) (**Supplementary Table 4**).

### 3.5 Secondary outcomes

Secondary outcome reporting was dominated by safety, manufacturing feasibility, and, where available, resection-to-infusion time. In melanoma RCTs, TIL therapy was generally feasible, with high manufacturing success. Dréno et al. reported 100% successful expansion, and no severe toxicities were noted (12). In the phase III randomized controlled trial (Rohaan et al., 2022), approximately 95.2% of patients assigned to TIL therapy received the final product, and only one non-receipt was attributed to manufacturing failure. Estimated resection-to-infusion time in that study was approximately 4–6 weeks (14).

The differential toxicity burden observed across trials was substantially influenced by variation in both the IL-2 regimen and the lymphodepletion chemotherapy regimen. High-dose bolus intravenous IL-2 (720, 000 IU/kg q8h), used in the majority of contemporary single-arm metastatic melanoma studies, was consistently associated with the highest incidence of grade ≥3 cytokine-related and systemic toxicities. In contrast, attenuated decrescendo IL-2 infusion schedules and low-dose subcutaneous IL-2 regimens were associated with more manageable toxicity profiles, with no grade 3–4 IL-2-related events reported in some low-dose cohorts (Ellebaek et al.) (21). These observations support ongoing efforts to optimise toxicity management through modification of IL-2 dosing strategies and highlight IL-2 regimen intensity as an important variable to be prospectively evaluated in future TIL trials.

Across non-randomized melanoma studies, toxicity patterns were largely consistent with the known effects of lymphodepleting chemotherapy and IL-2 administration. Hematologic toxicity, fever, hypotension, capillary leak-related manifestations, and transient constitutional symptoms were common. Neurotoxicity was reported in some early studies, particularly those using intensive IL-2 regimens (20, 25). Importantly, treatment-related mortality was uncommon but not absent; Baars et al. reported one treatment-related death due to pseudomonal septicemia (15). Manufacturing success rates were generally high in studies that reported them, often approaching 100%, with resection-to-infusion times typically spanning several weeks. Autoimmune toxicities, when reported, were usually limited and included vitiligo and occasionally uveitis, particularly in melanoma cohorts. In the randomized Rohaan study, grade ≥3 adverse events were markedly more frequent in the TIL arm than in the ipilimumab arm, reflecting the added toxicities of lymphodepletion and high-dose IL-2 (14) (**Supplementary Table 5**).

In non-melanoma studies, safety reporting was again centered on feasibility. Ovarian, head and neck, hepatocellular carcinoma, and osteosarcoma studies were generally small and early phase, with no major new safety signals beyond those expected from preparative chemotherapy and cytokine support.

### 3.6 TIL therapy as a combination regimen

Evidence on TIL therapy in combination regimens was derived from a limited number of early-phase and exploratory studies across multiple tumor types (**Supplementary Table 6**). In melanoma, several studies evaluated TIL therapy in combination with immune checkpoint inhibitors (49–52). Mullinax et al. reported feasibility of combining TIL therapy with ipilimumab, with 38% objective response rate (5/13) (50). Hall et al. evaluated TIL therapy in combination with nivolumab, reporting an ORR of 36% (4/11), with durable responses observed beyond 18 months in responding patients (49). In a recent exploratory study, L’Orphelin et al. reported an ORR of 75% (3/4) in patients receiving nivolumab in combination with TIL therapy (52). A randomized phase II study by Hasanov et al. evaluated TIL therapy with pembrolizumab and reported an overall ORR of 14% (2/14), with similar response rates across high-dose and low-dose IL-2 arms (51).

Beyond melanoma, combination strategies were also explored in other tumor types. In metastatic breast cancer, Zacharakis et al. reported objective responses in 3/6 patients (50%), including one complete response with ongoing remission beyond 66 months (53).

In gastrointestinal cancers, Lowery et al. reported ORR of 23.5% in patients treated with tumor-reactive TIL combined with pembrolizumab, compared with 7.7% with tumor-reactive TIL alone and 0% with bulk TIL (54). Other combinational approaches included the use of dendritic cell vaccines and cytokine-based regimens. Saberian et al. reported ORR of 50% in the TIL plus dendritic cell vaccine arm compared with 30% in the TIL-alone arm (55). In an earlier study, Queirolo et al. reported an ORR of 11% (2/19) with TIL combined with recombinant interferon-α (56). Across studies, combination regimens were predominantly evaluated in small cohorts and early-phase settings.

## 4 Discussion

Our principal quantitative finding, of a pooled ORR of 42% in melanoma, based on single-arm meta-analysis, derived from 18 cohorts with moderate heterogeneity, confirms sustained clinical activity of TIL therapy in metastatic melanoma (15–22, 24–33). Importantly, the present analysis extends beyond prior melanoma-focused syntheses by integrating evidence across randomized, non-randomized, and exploratory studies, and by incorporating safety, manufacturing feasibility, and emerging combination strategies. Compared with the most recent prior syntheses notably Mony & Veeraraghavan (2025) (7) and Martín-Lluesma et al. (2024) (57) the present analysis uniquely integrates: (i) operational feasibility data (manufacturing success and resection-to-infusion time) across all solid tumour types; (ii) systematic coverage of non-melanoma indications including NSCLC, NPC, HNSCC, HCC, osteosarcoma, and urothelial carcinoma; (iii) an updated evidence base through 31 December 2025, encompassing recent trials (McClelland et al., 2025 (37), Medina et al., 2025) (23); and (iv) synthesis of early-phase combination strategies across tumor types. The quantitative findings are supported by randomized and non-randomized evidence. The phase III trial comparing TIL therapy with ipilimumab demonstrated superior response rates and progression-free survival in advanced melanoma, while adjuvant studies reported numerically favorable survival outcomes (14). Across prospective single-arm studies, ORRs ranged from 0% to 75%, with multiple cohorts reporting response rates in the range of approximately 30%-56%. Durable complete responses were observed across studies, including long-term remissions extending beyond several years in selected cohorts. Taken together, these findings indicate that TIL therapy produces consistent and clinically meaningful antitumor activity across diverse clinical settings, including heavily pretreated and checkpoint inhibitor–refractory populations.

Subgroup analyses provide additional insights into determinants of response. Higher pooled ORR with tumor-reactive TIL products (pre-screened ex vivo for antigen-specific reactivity; n=4 cohorts, hypothesis-generating) (17, 18, 20, 26)than with bulk TIL (13, 15, 16, 19, 21, 27, 28, 31, 33), suggest that tumor-reactivity enrichment may influence therapeutic activity. In contrast, no significant differences were observed according to lymphodepletion status, although interpretation is limited by the small number of studies without lymphodepletion and the predominance of contemporary protocols incorporating standardized conditioning regimens. These findings highlight the complexity of disentangling the relative contributions of manufacturing strategies, preparative regimens, and patient selection to treatment outcomes.

Beyond melanoma, the evidence base was limited and heterogeneous but provides important context for the broader applicability of TIL therapy. Signals of activity were reported in several tumor types, including ovarian cancer, nasopharyngeal carcinoma, NSCLC, head and neck squamous cell carcinoma, and hepatocellular carcinoma. However, these findings were derived predominantly from small, early-phase, or non-randomized studies, and objective responses were less frequent than in melanoma (29, 42–45). In several tumor types, including colorectal cancer and osteosarcoma, responses were limited, although disease stabilization was observed in a proportion of patients (46, 47). Collectively, these findings suggest that the efficacy of TIL therapy is tumor-context dependent and may be influenced by tumor immunogenicity and microenvironmental factors.

Biological differences between tumor types likely underpin these observations. Melanoma is characterized by high tumor mutational burden, abundant neoantigen load, and a pre-existing inflamed tumor microenvironment, which collectively favor the expansion and persistence of tumor-reactive lymphocytes (58). In contrast, many non-melanoma solid tumors exhibit lower antigenicity, impaired T-cell infiltration, stromal exclusion, and metabolically restrictive tumor microenvironments. These features may limit the generation and *in vivo* functionality of TIL products (59, 60). The current findings therefore support a shift toward biomarker-driven strategies in TIL therapy, including stratification based on tumor mutational burden, baseline immune infiltration, interferon signaling signatures, and spatial immune architecture (51, 61, 62).

Combination strategies represent an emerging avenue to enhance TIL efficacy. Early-phase studies combining TIL therapy with immune checkpoint inhibitors demonstrated feasibility and variable clinical activity, with reported ORRs ranging from 14% to 75% across melanoma cohorts (49, 50, 52, 65). In non-melanoma tumors, combination approaches also showed activity in selected settings, including metastatic breast cancer and gastrointestinal malignancies (53, 54). Additional strategies, such as dendritic cell vaccination and cytokine-based combinations, have also been explored. These approaches aim to augment T-cell persistence and overcome tumor-mediated immunosuppression, although current evidence remains limited to small exploratory studies (55).

Subgroup analysis by lymphodepletion status demonstrated no statistically significant difference in pooled ORR between lymphodepleted and non-lymphodepleted cohorts. However, this analysis is substantially limited by the small number of included cohorts without lymphodepletion (n=3: Goedegebuure et al. (40), Khammari et al., 2015 (24), Ellebaek et al (21). partially), which had markedly lower patient numbers and predominantly comprised early adjuvant or pilot studies. Furthermore, the no-LDC cohorts differ from the Cy/Flu NMA cohorts not only in conditioning regimen but also in patient population (adjuvant vs. metastatic), TIL product type, and IL-2 schedule — making it impossible to attribute ORR differences to LDC status alone. The TBI-intensified cohorts of Rosenberg et al. (2011) (18) were included within the Cy/Flu stratum for this analysis, as no separate TBI subgroup analysis was feasible given that TBI and non-TBI patients were reported within the same study publication. The influence of LDC regimen on efficacy therefore remains a methodologically important but currently unresolvable question, and prospective trials designed to compare conditioning intensities with pre-specified efficacy endpoints are needed.

Safety findings were consistent across studies and largely attributable to the preparative regimen rather than the cellular product itself. Toxicities were dominated by lymphodepletion-associated cytopenias and IL-2-related adverse events, including capillary leak syndrome and constitutional symptoms. High-grade toxicities were common but predictable, and treatment-related mortality was uncommon. High-dose bolus intravenous IL-2 (720, 000 IU/kg q8h), used in the majority of contemporary single-arm metastatic melanoma studies, was consistently associated with the highest incidence of grade ≥3 cytokine-related toxicities. In contrast, attenuated decrescendo IL-2 infusion schedules and low-dose subcutaneous IL-2 regimens were associated with more manageable safety profiles, supporting ongoing efforts to optimise IL-2 dosing in future TIL protocols. These observations are consistent with prior institutional experiences and support ongoing efforts to optimize toxicity management, including modification of IL-2 dosing strategies.

Operational feasibility represents a critical determinant of clinical implementation. Manufacturing success rates were high across studies, frequently approaching 100% in contemporary cohorts, reflecting maturation of TIL production platforms. However, resection-to-infusion times of approximately 4–6 weeks remain a clinically relevant limitation, particularly in patients with rapidly progressive disease. Advances in manufacturing, including closed-system platforms and process optimization, will be essential to improve scalability and accessibility.

The interpretation of these findings must consider the underlying study design and quality. The evidence base was dominated by single-arm and non-randomized studies, with risk-of-bias assessments indicating moderate to serious limitations, particularly related to confounding and patient selection. In the absence of comparator arms, pooled ORR reflects treatment activity rather than comparative efficacy. In addition, substantial clinical and methodological heterogeneity across studies, including differences in lymphodepletion regimens, IL-2 dosing, TIL selection strategies, and prior therapies, likely contributed to variability in outcomes.

This study has several limitations. First, quantitative synthesis was restricted to melanoma cohorts, limiting generalizability across tumor types. Second, heterogeneity in study design and reporting precluded pooled analysis of survival outcomes. Third, publication bias cannot be excluded, particularly given the predominance of early-phase studies. Fourth, the non-melanoma evidence base remains limited and exploratory. fifth, geographic representation was skewed toward North America and Europe, with limited data from other regions. Sixth, formal meta-regression by IL-2 regimen type or lymphodepleting conditioning intensity was not feasible owing to confounding by TIL product type, patient selection, and treatment era across included studies; the impact of these regimen variables on efficacy cannot be disentangled from the current dataset and warrants prospective evaluation. In conclusion, this systematic review demonstrates that adoptive TIL therapy provides consistent and clinically meaningful antitumor activity in uncontrolled settings of melanoma, supported by both quantitative and qualitative evidence. In contrast, evidence in non-melanoma solid tumors remains limited and heterogeneous. Future research should focus on biomarker-driven patient selection, optimization of TIL manufacturing and conditioning strategies, and the development of rational combination approaches to extend the applicability of TIL therapy across solid tumors.

## Data Availability

The original contributions presented in the study are included in the article/**Supplementary Material**. Further inquiries can be directed to the corresponding authors.
