# Supplementary material for: Efficacy, safety, and characteristics of adoptive tumor-infiltrating lymphocyte therapy in solid tumours: a systematic review and meta-analysis
Source: Front Oncol. 2026 Jul 22;16:1859306. doi: 10.3389/fonc.2026.1859306 (PMC13437784; doi:10.3389/fonc.2026.1859306)
Supplement: Supplementary file 1 [file Supplementaryfile1.pdf]

Supplementary Table 1: Characteristics of included studies

· Author<br>· Country<br>· Year	· Study design<br>· Phase	· Intervention<br>· Control	· Participants enrolled<br>· Treated [IG/CG] n/n	· Cancer type<br>· Disease type	· Prior line of therapy<br>· Prior immunotherapy	Special population	· TIL Source<br>· TIL selection	Key regimen<br>· IL-2 Pre and post<br>· Cell dose*no. infusion	Lymphodepletion<br>· TBI	Outcome measured (primary and /or secondary outcomes)
**Melanoma**										
RCTs										
Dréno B[14]<br>France<br>2002	Randomized, open-label adjuvant trial<br>Phase II	TIL + IL-2<br>IL-2 alone	88<br>TIL + IL-2: 44<br>IL-2 alone (control): 44	Melanoma<br>Stage III melanoma (AJCC) with regional lymph-node involvement, no distant metastasis	Surgical lymph-node excision only (no prior systemic adjuvant therapy)<br>NR	Stratified by number of invaded lymph nodes (1 vs >1)	Invaded regional lymph nodes<br>Bulk TIL	Low-dose subcutaneous IL-2<br>6×10^6 IU/m²/day, 5 days/week for 2 weeks (× 2 cycles)<br>0.22×10^{10} – 3.34×10^{10} cells<br>Median 1.7×10^{10} cells * 2	No lymphodepletion<br>No TBI	Primary: Relapse-free survival (RFS)<br>Secondary: Overall survival (OS)
Khammari A[12]<br>France<br>2020	Open-label, randomized, two-arm clinical trial<br>Phase III	Autologous TIL + IL-2<br>Abstention (no adjuvant therapy)	81<br>49<br>TIL+IL-2: 26<br>Abstention (control): 23	Melanoma<br>Stage III melanoma (only one invaded lymph node, post-complete resection)	Prior adjuvant interferon-α allowed (≈ 47%)<br>Yes (low-dose IFN-α in some patients)	Minimal residual disease; adjuvant setting	Invaded metastatic lymph node<br>Bulk TIL	Subcutaneous IL-2 (6 million IU, day 1–5 and day 8–12)<br>$\geq 1 \times 10^9$ cells per infusion * 2	No lymphodepletion<br>No TBI	Primary: Disease-free survival (DFS)<br>Secondary: Overall survival (OS)<br>Safety / tolerance<br>Immunological correlates

Rohaan MW[13] Multinational 2022	Open-label RCT<br><br>Phase III	TIL + lymphodepletion + IL-2<br><br>Ipilimumab	168 (84/84)<br><br>~84/84	Melanoma Advanced (Stage IIIC–IV)	≤1 prior therapy Prior anti-PD-1 (majority refractory)	Advanced melanoma with resectable lesion	Bulk TIL	Cyclophosphamide + Fludarabine + TIL + HD IL-2 | High-dose IL-2 (720,000 IU/kg q8h)<br><br>≥5 × 10⁹ cells * single infusion	Yes 60mg/kg/day × 2 days Fludarabine 25 mg/m²/day × 5 days<br>No TBI	Primary: PFS Secondary: ORR, OS, CR, Safety
n-RCTs Single arm trials										
Baars JW[22]<br><br>Netherlands<br><br>1992	Pilot, single-arm<br><br>interventional study<br><br>Phase I	Autologous expanded TIL + IL-2 after cyclophosphamide<br><br>NR	4<br><br>4	Melanoma Metastatic melanoma	Radiotherapy (2 patients)<br>Chemotherapy (dacarbazine; 1 patient)<br>Immunotherapy (all patients: IL-2, IFN-γ ± ASI)<br>Yes (100%)	Heavily pre-treated metastatic melanoma	Resected metastatic lesions<br>Lymph node, cutaneous, or subcutaneous metastases<br><br>Bulk TIL (no tumor-reactivity selection)	IL-2 18 × 10⁶ IU/m²/day × 5 days<br>Continuous infusion (3 patients)<br>Bolus regimen (1 patient)<br>1.0 × 10¹⁰ – 5.56 × 10¹⁰ cells (total per patient) * 2–3 infusions per patient	Cyclophosphamide 1 g/m² IV (single dose, day −2)<br>No TBI	Primary: Feasibility and toxicity<br>Secondary: NR
P S Goedegebuure[42]<br><br>USA<br><br>1995	Single-arm pilot interventional study<br>Pilot	Autologous TIL + moderate-dose IL-2	26<br><br>26	Melanoma (n=18), Renal cell carcinoma (n=8)<br>Metastatic / advanced	Refractory to standard therapy<br><br>None (IL-2-naïve by eligibility)	Heavily pretreated metastatic disease	Fresh tumor biopsies (LN, lung, kidney, soft tissue)<br>Bulk polyclonal TIL	Moderate-dose IL-2 (30,000 IU/kg IV q8h; up to 28 doses)<br>≥1 × 10¹⁰ TIL (mean ~6 × 10¹⁰) * 3	No lymphodepletion<br><br>No TBI	Primary: Tolerance and antitumor efficacy<br>Secondary: NR

								infusions (days 2, 4, 6)		
Dudley ME[23]<br><br>USA<br><br>2005	Single-arm interventional clinical study<br><br>Phase II–like (not formally labeled)	Autologous TIL + lymphodepletion + high-dose IL-2	35<br><br>35	Melanoma Refractory metastatic melanoma, Stage IV	NR<br><br>High-dose IL-2 in 34/35 patients	Brain, liver, lung, and bone metastases included	Resected metastatic lesions<br><br>Tumor-reactive bulk TIL	High-dose IL-2 (720,000 IU/kg IV q8h)<br><br>Mean 6.3 × 10^{10} cells (range 1.1–16.0 × 10^{10}) * 1 (some patients received re-treatment)	Cyclophosphamide + Fludarabine (non-myeloablative)<br><br>No TBI	Primary: Objective tumor response<br>Secondary: Safety
Rosenberg SA[24]<br><br>USA<br><br>2011	Prospective non-randomized Phase II	Autologous TIL + IL-2 after lymphodepletion<br><br>NR	93<br><br>93	Metastatic melanoma Advanced, refractory metastatic melanoma	Median prior systemic therapies: 2<br>95% progressed after ≥1 prior systemic treatment<br><br>IL-2: 83%<br>Interferon-α: 56%<br>Anti-CTLA-4: 12%	Heavily pretreated; 86% with visceral metastases (M1b/M1c)	Surgically resected metastatic melanoma lesions<br><br>Tumor-reactive TIL selected by IFN-γ release assay	High-dose IL-2 (720,000 IU/kg IV q8h to tolerance)<br>Mean 5.5–6.5 ×10^{10} cells (range categories reported) * 1 (2 patients received a second infusion)	Cyclophosphamide 60 mg/kg × 2 days<br>Fludarabine 25 mg/m² × 5 days<br>None: 43 patients<br>2 Gy TBI: 25 patients<br>12 Gy TBI: 25 patients	Primary: Objective response rate (RECIST)<br>Secondary: NR
Radvanyi LG[25]<br><br>USA<br><br>2012	Prospective Phase II clinical trial (non-randomized component analyzed)	Autologous expanded TIL + lymphodepletion + HD IL-2<br><br>NR	31<br><br>31	Melanoma Advanced/metastatic melanoma (Stage IIIc–IV; M1a–M1c)	Most patients had ≥2 prior systemic therapies<br>Prior IL-2, IFN-α, GM-CSF allowed<br>No prior ipilimumab	Heavily pre-treated metastatic melanoma; small brain metastases allowed (≤1 cm)	Surgically resected metastatic melanoma lesions<br>Bulk TIL (no selection for	High-dose IL-2 (720,000 IU/kg IV q8h)<br>Two cycles (most patients)	Cyclophosphamide 60 mg/kg × 2 days<br>Fludarabine 25 mg/m² × 5 days<br>No TBI	Primary: Objective tumor response using immune-related response criteria (irRC)<br><br>Secondary: Biomarker correlations (TIL

							tumor reactivity)	8×10^9 – 150×10^9 cells * 1		phenotype, clonotype persistence, telomere length)
Pilon-Thomas S[26]<br><br>USA<br><br>2012	Single-arm pilot clinical trial with pre-trial feasibility cohort<br><br>Early phase	Non-myeloablative lymphodepletion → TIL infusion → high-dose IL-2<br>NR	19<br><br>13	Metastatic melanoma Stage IV (predominantly M1c)	Surgery, chemotherapy, immunotherapy, radiation, targeted therapy (multiple prior lines per patient; detailed per-patient in Table II)<br>Yes (multiple patients)	Heavily pre-treated metastatic melanoma	Surgically resected metastatic melanoma lesions (subcutaneous, LN, lung, bowel, intramuscular)<br>Fragment-derived TIL selected based on IFN-γ secretion against autologous or HLA-matched tumor	High-dose IL-2 720,000 IU/kg IV q8h to tolerance (≤5 days)<br>Median 5.2×10^{10} cells<br>Range approx. $2.0–11.0 \times 10^{10}$ * 1	Cyclophosphamide 60 mg/kg × 2 days<br>Fludarabine 25 mg/m² × 5 days<br>No TBI	Primary: Feasibility and tumor response<br><br>Secondary: Toxicity
Ellebaek E[27]<br><br>Denmark<br><br>2012	Single-arm pilot feasibility study Pilot (Phase I/feasibility)	Autologous TIL therapy ± lymphodepletion ± cytokine support (specify details)<br><br>None (single-arm study)	11<br><br>6	Malignant melanoma Metastatic	Heavily pretreated (High-dose IL-2 ± IFN-α; some received DC vaccine, ipilimumab)	Patients with prior brain metastases (resected before inclusion)	Metastatic lesions (subcutaneous nodules or lymph nodes)<br><br>Bulk TIL cultures; selected for growth and phenotype	Low-dose subcutaneous IL-2 (2 MIU/day × 14 days)<br><br>Mean 2.6×10^{10} cells (range $3.4–74.7 \times 10^9$) * Single infusion	Cyclophosphamide + Fludarabine<br><br>No TBI	Primary: Feasibility and safety, with exploratory efficacy<br>Secondary: Clinical response, Time to progression (TTP), Overall survival (OS), Immunological correlates

Besser MJ[28]<br><br>Israel<br><br>2013	Prospective, single-arm interventional (intent-to-treat analysis)<br><br>Phase II	Autologous unselected (“young”) TIL + HD-IL-2<br><br>NR	80<br><br>57	Melanoma Metastatic melanoma, Stage IV (M1a–c); 79–80% M1c	All patients received prior systemic therapy<br><br>IL-2-based therapy 95%; ipilimumab 23%	CNS metastases allowed (19% at baseline)	Surgically resected metastatic lesions (lung, LN, SQ, visceral, brain)<br><br>Unselected / minimally cultured (“young”) TIL	High-dose IL-2 (720,000 IU/kg IV bolus)<br><br>Mean 5.2 × 10^{10} cells (range approx. $1×10^{10}$–$1×10^{11}$) * Single infusion	Cyclophosphamide + Fludarabine (non-myeloablative)<br>No TBI	Primary: Objective response rate (ITT and treated)<br><br>Secondary: PFS, OS
EUCTR_2014-001419-38 Denmark[40]	Single-arm interventional clinical trial<br><br>Not explicitly stated (feasibility/toxicity-focused; early phase)	Vemurafenib pretreatment → lymphodepletion → TIL infusion → IL-2<br><br>NR	13<br><br>12	Metastatic melanoma BRAF-mutated metastatic melanoma	NR<br><br>NR	BRAF-mutated melanoma only	Surgically resected metastatic melanoma lesion<br><br>Young TIL method	Interleukin-2 decrescendo regimen<br><br>$1×10^9$ – $2×10^{11}$ cells * 1	Cyclophosphamide 60 mg/kg × 2 days, Fludarabine 25 mg/m² × 5 days<br>No TBI	Primary: Toxicity and feasibility (CTCAE v4.0)<br><br>Secondary: Objective response rate (RECIST 1.1), Complete response rate: 8.3%, Partial response rate: 66.7%, Disease control rate (CR+PR+SD): 100%, Response criteria: RECIST 1.1
Khammari A[29]<br><br>France<br><br>2015	Open-label, monocentric phase I/II clinical trial	Autologous TIL + subcutaneous IL-2 + intratumoral TG1042	18<br><br>13	Melanoma Unresectable stage IIIc / stage IV	≥1 prior therapy in 13/18 patients<br><br>IFN-α, vaccines, and chemotherapy in several patients)	Cutaneous and nodal metastatic melanoma; no brain/bone mets	Cutaneous melanoma metastases Bulk minimally cultured TIL	Subcutaneous IL-2 (6 MIU/day × 10 days)<br>1.66 × 10^9 – 22.2 × 10^9 TIL * 2 infusions planned; 11 pts	No lymphodepletion<br><br>No TBI	Primary: Safety of combined therapy<br><br>Secondary: NR

								received 2, 2 pts only 1		
AndersenR[31]<br><br>Denmark<br><br>2016	Phase I/II, single-arm interventional trial	TIL infusion after lymphodepletion + attenuated IL-2 decrescendo regimen<br><br>NR	25<br><br>25	Melanoma Treatment-refractory metastatic melanoma (AJCC III unresectable / IV)	1–4 prior systemic therapies (median 2)<br>Prior IL-2: 96%<br>Prior ipilimumab: 80%	Heavily pretreated, salvage-setting melanoma<br>Elevated LDH in 60%<br>M1c disease in 84%	Surgically resected metastatic melanoma lesions<br><br>Young TIL (bulk; no tumor-reactivity pre-selection)	IL-2 continuous decrescendo regimen<br>Median total dose 112 MIU (≈ 93% of planned)<br>Median 9.8 × 10^{10} cells<br>Range 6.1–2.0 × 10^{11} * 1	Cyclophosphamide 60 mg/kg/day × 2 days<br>Fludarabine 25 mg/m²/day × 5 days<br>No TBI	Primary: Safety and feasibility<br><br>Secondary: ORR, PFS, OS, immunologic correlates
Chandran SS[32]<br><br>USA<br><br>2017	Single-arm, open-label, two-stage Phase II clinical trial	Autologous TIL + lymphodepletion + high-dose IL-2<br>NR	27 (underwent metastasectomy)<br>Sample size (Treated): 21<br>Sample size (Evaluable for response): 20	Melanoma Metastatic uveal (ocular) melanoma	2 prior systemic regimens: 57%<br>Anti-CTLA-4 and/or anti-PD-1: 43%	Predominantly liver-metastatic disease (95%)	Surgically resected metastatic lesions (liver and extra-hepatic)<br>Bulk TIL with functional tumor-reactivity testing	High-dose IL-2 (720,000 IU/kg IV q8h; median 5 doses)<br><br>Median 85 × 10^9 cells (range 17–138 × 10^9) * 1 (2 patients received re-treatment)	Cyclophosphamide 60 mg/kg × 2 days<br>Fludarabine 25 mg/m² × 5 days<br>No TBI	Primary: Objective response rate (RECIST v1.0)<br><br>Secondary: Toxicity
Forget MA[33]<br><br>USA<br><br>2018	Prospective, non-randomized, open-label clinical trial Phase II	Autologous TIL ACT + IL-2 after lymphodepletion<br><br>NR	74<br><br>74	Melanoma Metastatic melanoma (cutaneous, mucosal, uveal)	Allowed (surgery, chemo, targeted, immunotherapy)<br>Checkpoint-naïve: 43<br>Anti-CTLA-4 exposed: 30<br>Anti-PD-1 exposed: 9	Brain metastases ≤1 cm allowed if stable	Resected metastatic melanoma lesions<br><br>Bulk TIL; no pre-selection for tumor reactivity	High-dose IL-2 (720,000 IU/kg q8h, up to 15 doses)<br>Second IL-2 course ~day 21<br><br>Variable	Cyclophosphamide 60 mg/kg (days −7, −6)<br>Fludarabine 25 mg/m² (days −5 to −1)<br>No TBI	Primary: Best overall response (irRC), OS, PFS<br><br>Secondary: Toxicity

					(mostly with CTLA-4)			(higher dose correlated with response) * 1		
Nguyen LT[34]<br><br>Canada<br><br>2019	Prospective single-arm Phase II clinical trial	Lymphodepletion → TIL infusion → low-dose subcutaneous IL-2<br>NR	12<br><br>12	Melanoma Unresectable metastatic melanoma	Heavily pretreated; 11/12 received ≥1 prior systemic therapy<br>Ipilimumab: 10/12<br>Anti-PD-1 (nivolumab/pembrolizumab): 9/12<br>Combination Ipi+Nivo: 2/12	Brain metastases allowed (treated/stable): 4 patients	Surgically resected metastatic lesions (LN and non-LN sites)<br><br>Bulk TIL<br>No mandatory tumor-reactivity or CD8 preselection	Low-dose subcutaneous IL-2<br>125,000 IU/kg/day<br>Max 9–10 doses over ~12 days<br>Mean received: 6.8 doses<br><br>Mean: 1.12 × 10^{11} cells<br>Range: 5.5 × 10^{10} – 1.6 × 10^{11} * 1	Cyclophosphamide 60 mg/kg/day × 2 days<br>Fludarabine 25 mg/m²/day × 5 days<br><br>No TBI	Primary: Objective response rate (RECIST v1.1)<br>Secondary: Safety (CTCAE v4.0)<br>Immune correlates / TIL persistence
NCT01701674[41]<br><br>USA	Open-label, single-arm pilot interventional trial pilot/feasibility study	Ipilimumab → lymphodepletion → TIL infusion → high-dose IL-2<br>NR	13<br><br>12	Melanoma Unresectable metastatic melanoma (stage III/IV)	Prior systemic therapy allowed<br>No prior ipilimumab permitted<br>Prior BRAF inhibitor allowed if BRAF V600-mutant<br>No prior anti-CTLA-4	Patients eligible for high-dose IL-2 and ACT	Surgically resected metastatic melanoma lesion<br>Bulk TIL	Ipilimumab → lymphodepletion → TIL infusion → high-dose IL-2<br>High-dose IL-2 (bolus IV, 3× daily for ~3–5 days)<br>NR * 1	Cyclophosphamide (IV, 2 days)<br>Fludarabine (IV, 5 days)<br>No TBI	Primary: Dose-limiting toxicity (DLT)<br>Feasibility of combined therapy<br>Secondary: Median PFS
van den Berg[35] JH<br>Netherlands	Prospective Phase I/II feasibility study<br>Phase I/II	Autologous expanded "young" TIL +	13<br>10 (3 progressed before infusion)	Melanoma<br>Metastatic cutaneous	Chemotherapy (DTIC, temozolomide)<br>Targeted therapy	Heavily pre-treated; anti-PD-1–naïve at infusion	Surgically resected melanoma metastases	Autologous expanded "young" TIL + lymphodepletio	Cyclophosphamide 60 mg/kg/day × 2 days	Primary: Feasibility and clinical activity of TIL therapy at NKI<br>Secondary: Neoantigen-

2020		lymphodepletion + HD IL-2<br>NR		melanoma (Stage IIIc / IV)	(vemurafenib)<br>Vaccines (DC / peptide)<br>Yes (ipilimumab, IFN-α); anti-PD-1 naïve		Bulk "young" TIL (no tumor-reactivity selection)	n + HD IL-2<br>High-dose IL-2 600,000 IU/kg IV q8h<br>Median 3.5 doses (range 1–9)<br>Mean: 12.8 × 10^{10} cells<br>Range: 1.7–19.6 × 10^{10} * 1	Fludarabine 25 mg/m²/day × 5 days<br>No TBI	specific T-cell reactivity<br>Long-term follow-up and persistence
Hirai I[36]<br><br>Japan<br><br>2021	Open-label, single-arm feasibility (pilot) study<br><br>Pilot / Phase I-equivalent (feasibility)	ACT-TIL (bulk autologous TIL infusion + Cy/Flu + IL-2)<br>None (single-arm study)	4<br><br>3	Melanoma<br>Metastatic melanoma refractory to immune-checkpoint inhibitors	Heavily pretreated (≥3 prior lines)<br><br>anti-PD-1 ± anti-CTLA-4	Acral, mucosal, and superficial spreading melanoma; post-ICI failure	Resected metastatic tumor tissue<br>Bulk TILs (no marker-based enrichment)	Low-dose IL-2 (72,000 IU/kg IV q8h, max 15 doses)<br><br>Mean 6.38 × 10^{10} cells (range 2.7–10.2 × 10^{10}) * Single dose	Cyclophosphamide + fludarabine (non-myeloablative)<br><br>No TBI	Primary: Feasibility (completion without unacceptable toxicity)<br>Secondary: Safety, ORR, PFS, OS
Sarnaik AA[37]<br><br>Multinational (USA, Europe)<br><br>2021	Phase II, open-label, single-arm trial	Lifileucel (LN-144) + lymphodepletion + IL-2<br><br>NR	78 underwent tumor resection<br>66 (full analysis set)	Melanoma<br>Unresectable or metastatic melanoma (Stage IIIC–IV)	Mean 3.3 prior therapies (range 1–9)<br><br>Anti-PD-1 / PD-L1: 100%<br>Anti-CTLA-4: 80%	Heavily pretreated, post-ICI melanoma<br><br>Primary anti-PD-1 refractory subset: 64%	Surgically resected metastatic melanoma lesions (skin, LN, liver, lung, etc.)<br><br>Bulk "young TIL" (no tumor-reactivity pre-selection)	High-dose IL-2 (600,000 IU/kg IV q8–12 h, up to 6 doses)<br><br>1 × 10^9 – 150 × 10^9 cells<br>Mean: 27.3 × 10^9 cells * 1	Cyclophosphamide 60 mg/kg/day × 2 days<br>Fludarabine 25 mg/m²/day × 5 days<br>No TBI	Primary: Objective Response Rate (ORR) by investigator-assessed RECIST v1.1<br><br>Secondary: NR

Chesney J[38]<br>Multinational (USA, Germany, UK)<br>2022	Prospective, single-arm, open-label, pooled cohort analysis<br>Phase II	Lifileucel TIL + lymphodepletion + IL-2<br>NR	189<br>153	Melanoma Unresectable or metastatic melanoma (stage IIIC–IV)	Median 3 (range 1–9)<br>Anti-PD-1/PD-L1: 100%<br>Anti-CTLA-4: 81.7%<br>Anti-PD-1 + anti-CTLA-4: 53.6%	Heavily pretreated, post-ICI ± post-BRAF/MEK<br>54.2% primary refractory to PD-1 therapy	Resected melanoma lesions (≥1.5 cm)<br>Bulk polyclonal TIL (no antigen-specific selection)	High-dose IL-2 (up to 6 doses)<br>Median 21.1 × 10^9<br>Range 1.2 × 10^9 – 99.5 × 10^9 * 1	Cyclophosphamide + Fludarabine (non-myeloablative)<br>No TBI	Primary: Independent Review Committee (IRC)-assessed ORR (RECIST v1.1)<br>Secondary: DOR, PFS, OS, safety
König D[39]<br>Switzerland<br>2024	Interventional, single-arm<br>Phase I	NR<br>NR	11<br>9	Cutaneous melanoma<br>Unresectable stage III / IV	Median 3 (range 1–5)<br>100% prior anti-PD-1	Heavily pre-treated; 1 patient with treated brain metastasis	Metastatic tumor lesion<br>Bulk TIL ($CD3^+$ polyclonal)	Low-dose IL-2 (125 000 IU/kg for 10 days)<br>Median 66.25 × 10^9 $CD3^+$ cells (range 50–84) * Single	Cyclophosphamide + Fludarabine<br>No TBI	Primary: Safety<br>Secondary: ORR, PFS, OS
Retrospective studies										
Saint-Jean [21]<br>France<br>2018	Retrospective observational study<br>NR	Autologous TIL + low-dose IL-2<br>NR	10<br>10	Melanoma Advanced/unresectable	≥2 prior treatments in 9/10 patients<br>Anti-CTLA-4 (ipilimumab) in 4 patients	Heavily pretreated; elderly patients included	Cutaneous or subcutaneous metastasis<br>Bulk polyclonal TIL	IL-2<br>Low-dose subcutaneous IL-2<br>0.23 × 10^9 – 22.9 × 10^9 cells * 2 per patient (1 patient received 4)	No lymphodepletion<br>No TBI	Primary: Efficacy and safety<br>Secondary: OS,PFS
Mehta GU[19]<br>USA<br>2018	Retrospective cohort analysis of prospectively treated clinical-	Lymphodepletion → TIL infusion → high-dose IL-	144 (all received TIL ACT)<br>M1c, no brain	Cutaneous melanoma<br>Stage M1c	Heavily pre-treated (exact number NR)<br>Some patients	Patients with treated or untreated	Resected melanoma lesions<br>Fragment-	High-dose IL-2 (720,000 IU/kg q8h as tolerated)	Cyclophosphamide 60 mg/kg × 2 days	Primary: Systemic and intracranial tumor response; PFS; OS

	trial patients Phase II-type ACT programs (multiple protocols pooled)	2: NR	metastases: n = 111 M1c, treated brain metastases: n = 15 M1c, untreated brain metastases: n = 18	metastatic melanoma	(not stratified; NR)	melanoma brain metastases	derived bulk TILs	Cell dose infused (median, ×10⁹): No brain mets: 69 (9–108), Treated brain mets: 52 (31–137), Untreated brain mets: 51 (10–109) * 1	Fludarabine 25 mg/m² × 5 days 39% overall (200 cGy or 1,200 cGy)	Secondary: Toxicity
McClelland PH[20] USA 2025	Retrospective cohort analysis of prospectively treated patients Phase I/II protocols pooled	ACT-TIL (acral melanoma) Non-acral melanoma cohort (internal comparator)	442 (cutaneous melanoma ACT-TIL cohort) 30 acral melanoma patients	Acral lentiginous melanoma Metastatic/unresectable	NR CTLA-4: 17%; PD-1/PD-L1: 7%	Low TMB melanoma; sun-shielded subtype	Bulk TIL (some CD8-enriched / alternate TIL protocols)	High-dose IL-2 (IV aldesleukin) Median 44 × 10⁹ cells (IQR 23–55) * Single infusion	Cyclophosphamide + Fludarabine (non-myeloablative) No TBI	Primary: Objective response to ACT-TIL Secondary: PFS, OS, and duration of response
**Carcinoma Ovary**										
non-RCTs (Two-arm studies)										
Fujita K[18] Japan 1995	Non-randomized controlled clinical trial (TIL vs chemotherapy-only control) Not stated (pre-phase / early translational clinical study)	Adoptive transfer of cryopreserved autologous TILs Chemotherapy only	24 disease-free patients post-chemotherapy TIL group: 13 Control group: 11	Ovarian cancer Advanced epithelial ovarian cancer, II–IV	Primary surgery + cisplatin-containing chemotherapy (3–5 cycles) NR	Patients with no detectable disease after chemotherapy (minimal residual disease setting)	Primary ovarian tumor tissue (resected at surgery) Bulk TIL (no tumor-reactivity selection)	IL-2 used only for culture, not given to patients >1 × 10¹⁰ cells (after re-expansion) * 1	No lymphodepletion No TBI	Primary: Overall survival and disease-free survival Secondary: Toxicity

non-RCTs (Single-arm studies)										
EUCTR_2015-000530-30<br>Denmark	Single-arm interventional pilot study<br>early phase	TIL infusion following lymphodepletion + IL-2<br><br>NR	6<br><br>6	Ovarian cancer<br>Advanced epithelial ovarian cancer, Stage IIIc: 4, Stage IV: 2	2–6 prior regimens (median 3)<br><br>NR	Heavily pre-treated recurrent ovarian cancer	Surgically resected ovarian tumor tissue<br><br>Bulk TIL (no tumor-reactivity preselection reported)	IL-2 continuous decrescendo infusion (≈5 days)<br><br>5×10^{9} – 2×10^{11} cells * 1	Cyclophosphamide (days −7 to −1), Fludarabine (days −7 to −1)<br>No TBI	Primary: Tolerability and feasibility (CTCAE v4.0)<br><br>Secondary: NR
Kverneland[42]<br><br>Denmark<br><br>2020	Prospective, single-arm pilot study<br>Early phase (Phase I/feasibility)	Autologous ACT-TIL combined with checkpoint inhibitors<br><br>None (Single-arm study).	7<br><br>6	Ovarian cancer<br>High-grade serous ovarian carcinoma	Heavily pretreated (median 4 lines)<br>No prior ACT; some prior systemic therapy	Platinum-resistant disease	Resected metastatic lesions<br><br>Unselected bulk TILs	Low-dose IL-2 (s.c.)<br><br>Median 8.47 × 10^{10} cells * Single infusion	Cyclophosphamide + Fludarabine<br><br>No TBI	Primary: Safety and feasibility<br>Secondary: Radiologic response (RECIST 1.1), Immune monitoring
**Carcinoma Colon**										
non-RCTs (Two-arm studies)										
Gardini A[17]<br><br>Italy<br><br>2004	Prospective, open-label, non-RCT<br><br>NR	Adjuvant adoptive immunotherapy with autologous TIL + IL-2 after radical liver resection.<br><br>Standard postoperative	47 (22/25) Immunotherapy arm: 14 (received TIL + IL-2)/ Control arm: 22	Colorectal cancer<br>Liver-limited colorectal metastases after radical hepatic resection	Surgical resection; no prior systemic immunotherapy/ chemotherapy	NR	Surgically resected colorectal liver metastases<br>Bulk TIL (no antigen-specific or CD8 selection)	Continuous IV IL-2 (5 days → 1-week break → 4.5 days), Monthly SC IL-2 maintenance for 6 months<br><br>1.18–7.04 × 10^{10} TIL	No lymphodepletion<br><br>No TBI	Primary: Overall survival (OS), Disease-free survival (DFS)<br><br>Secondary: Toxicity

		management ± conventional 5-FU–based chemotherapy						(median 3.24 × 10^{10}) * One infusion, Second infusion possible if high TIL yield achieved		
non-RCTs (Single-arm studies)										
Amaria R[43]<br><br>USA<br><br>2024	Non-randomized, open-label, multi-cohort clinical trial Phase II	Autologous ex vivo expanded TIL + lymphodepletion + IL-2<br><br>NR	27<br>16: CRC: 8<br>PDAC: 5<br>Ovarian: 3	Colorectal cancer Pancreatic ductal adenocarcinoma Ovarian cancer Recurrent or refractory metastatic disease	Median 2 (range 1–8)<br><br>Yes: 2/16 (12.5%) (1 OVCA, 1 CRC; pembrolizumab	Heavily pre-treated, checkpoint-refractory solid tumors	Surgically resected metastatic tumor tissue<br><br>Bulk TIL (no tumor-reactivity prescreening)	High-dose IL-2 (600,000 IU/kg IV, up to 6 doses)<br>Median 7.6 × 10^{10}<br>Range 2.03 × 10^{10} – 1.5 × 10^{11} * 1	Cyclophosphamide 60 mg/kg (or 30 mg/kg if heavily pretreated) Fludarabine 25 mg/m² (days −5 to −1)<br>No	Primary: Objective response rate (ORR) at 12 weeks (RECIST 1.1)<br><br>Secondary: DCR, PFS, OS, duration of response, safety
**NSCLC**										
RCTs										
Ratto GB [15]<br><br>Italy<br><br>1996	RCT<br><br>Phase II/III	ACT using autologous bulk TIL plus IL-2 following surgical resection (± RT)<br><br>Standard postoperative management	131<br>57/56	Non-small cell lung carcinoma (NSCLC)<br><br>Resected Stage II, IIIa, IIIb	Treatment-naïve<br><br>NO	Non-specified	Resected tumor tissue (tumor + surrounding lung)<br><br>Bulk TILs	Low-dose IL-2, subcutaneous, ~1–3 × 10^6 IU/day (3 months)<br><br>Median 20 × 10^9 (range 4–70 × 10^9) * Single dose	No lymphodepletion<br><br>No TBI	Primary: OS<br>Secondary: Local recurrence, Distance recurrence, toxicity

		(surgery ±<br>chemotherapy<br>/radiotherapy),<br>without TIL<br>or IL-2								
**Carcinoma Nasopharynx**										
RCTs										
Liang YJ [16]<br><br>China<br><br>2023	Open-label<br>RCT<br><br>Phase 2	CCRT+TIL<br><br>CCRT	78/78<br><br>78/49	Ca Nasopharynx<br><br>locoregionally<br>advanced NPC<br>(clinical stage of<br>III–IVb)	Treatment-naïve<br><br>NO	EBV DNA–<br>selected high-<br>risk NPC	Biopsied NPC<br>tumors<br><br>Bulk TILs	Low-dose IL-2<br>200,000 IU IM<br>daily × 2 weeks<br><br>> 1 × 109<br>*single dose	No<br>lymphodeplet<br>ion<br><br><br>No TBI	Primary: 3-year PFS<br><br><br>Secondary: OS, DMFS,<br>LRFS, Tumor Response<br>Rates, Safety,<br>Compliance
**Head and Neck SCC**										
non-RCTs (Single-arm studies)										
NCT03083873[4]<br>[4]<br><br><br>USA	Prospective,<br>open-label, non-<br>randomized,<br>single-arm,<br>multicohort<br>interventional<br>study<br><br><br>Phase II	LN-145 / LN-<br>145-S1<br>autologous<br>TIL + NMA<br>lymphodepleti<br>on + IL-2<br>NR	64<br>Cohort 1 (Gen<br>1, non-<br>cryopreserved):<br>8<br>Cohort 2 (Gen<br>2,<br>cryopreserved):<br>17<br>Cohort 3 (Gen<br>3,<br>cryopreserved):<br>16<br>Total infused:<br>41	Head and neck<br>cancer<br>Recurrent and/or<br>metastatic<br>squamous cell<br>carcinoma of the<br>head and neck<br>(HNSCC)	1–3 prior<br>systemic<br>therapies<br>(immunotherapy<br>and/or<br>chemotherapy<br>required)<br>Allowed and<br>common (PD-1<br>inhibitors<br>permitted)	PV-positive and<br>HPV-negative<br>HNSCC<br>included	Surgically<br>resected tumor<br>lesion<br>Bulk autologous<br>TIL (no tumor-<br>reactivity pre-<br>selection<br>reported)	IL-2<br><br><br>NR (not<br>disclosed in<br>registry) * 1	Non-<br>myeloablativ<br>e<br>cyclophospha<br>mide +<br>fludarabine<br>regimen<br><br><br>No TBI	Primary: Objective<br>response rate (ORR) per<br>RECIST v1.1 (CR + PR)<br>up to 24 months<br><br><br>Secondary: Toxicity
**Hepatocellular carcinoma**										

non-RCTs (Single-arm studies)										
Jiang SS[30]<br><br>China<br><br>2015	Single-arm, prospective clinical trial Phase 1	Autologous TIL adoptive cell therapy<br><br>None (no control arm)	17<br><br>15	Hepatocellular carcinoma (HCC) Primary HCC, post-surgical (no distant metastasis)	NR<br><br>NO	HBV-positive HCC (100%); some with cirrhosis	Resected primary HCC tumor tissue<br><br>Bulk TILs	0.3×10^9 to 3×10^9 cells per infusion * 1–2 infusions	No lymphodepletion<br><br>No TBI	Primary: Safety<br><br>Secondary: Immunologic response and clinical outcome
**Urothelial carcinoma**										
non-RCTs (Single-arm studies)										
Sherif A[46]<br><br>Sweden<br><br>2010	Open-label feasibility early phase	Autologous tumor-reactive T-helper cells expanded from sentinel / metinel lymph nodes<br>NR	12<br>6/6	Urothelial carcinoma of the urinary bladder Advanced / metastatic UBC, T2–T4b, N1–2, M0–M1 / MX	No prior chemotherapy or radiotherapy (explicitly stated)<br>NR	Metastatic disease; some patients with liver/bone metastases	Tumor-draining lymph nodes Sentinel nodes (SN) and metinel nodes (MN)<br>Tumor-reactive $CD4^+$ T-helper cells (Th1 biased)	Autologous tumor-reactive T-helper cells expanded from sentinel / metinel lymph nodes<br>None (IL-2 only used ex vivo)<br><br>29–704 $\times 10^6$ cells<br>Mean ≈ 313 $\times 10^6$ cells * 1 infusion (1 patient received a second infusion)	No lymphodepletion<br><br>No TBI	Primary: Feasibility and safety of MN-based adoptive T-cell therapy<br><br>Secondary: NR
**Osteosarcoma**										

non-RCTs (Single-arm studies)										
Wang C[45]<br><br>China<br><br>2022	Exploratory, single-arm clinical trial<br>Phase I	Autologous TIL + lymphodepletion + high-dose IL-2<br>NR	12<br><br>11	Osteosarcoma Metastatic (lung ± other sites)	All progressed after standard chemotherapy; many received targeted therapy; 3 received prior ICI<br><br>Subset received PD-1 inhibitors	Adolescents and young adults	Lung metastases, abdominal LN/wall metastases<br>Bulk TIL	High-dose IV IL-2 (100,000–200,000 U/kg)<br>10^9–10^{11} total cells * Single infusion	Fludarabine + cyclophosphamide<br>No	Primary: Safety and tolerance<br>Secondary: OS,PFS

RCT, randomized controlled trial; n-RCT, non-randomized controlled trial; NR, not reported; IG, intervention group; CG, control group; TIL, tumor-infiltrating lymphocytes; ACT, adoptive cell therapy; IL-2, interleukin-2; HD IL-2, high-dose interleukin-2; IFN-α, interferon-alpha; IFN-γ, interferon-gamma; GM-CSF, granulocyte-macrophage colony-stimulating factor; ASI, active specific immunotherapy; LN, lymph node; SQ, subcutaneous; IV, intravenous; IM, intramuscular; Cy, cyclophosphamide; Flu, fludarabine; TBI, total body irradiation; NMA, non-myeloablative; AJCC, American Joint Committee on Cancer; NSCLC, non-small cell lung cancer; HNSCC, head and neck squamous cell carcinoma; HCC, hepatocellular carcinoma; NPC, nasopharyngeal carcinoma; RCC, renal cell carcinoma; CRC, colorectal cancer; PDAC, pancreatic ductal adenocarcinoma; OVCA, ovarian cancer; UBC, urothelial bladder cancer; CNS, central nervous system; EBV, Epstein–Barr virus; HPV, human papillomavirus; HBV, hepatitis B virus; TMB, tumor mutational burden; IU, international units; MIU, million international units; CD, cluster of differentiation; HLA, human leukocyte antigen.

Supplementary Table 2: Risk of bias assessment of retrospective studies

Study	Year	Tumor_type	Study_type	Selection_bias	Confounding	Exposure_classification	Outcome_assessment	Reporting	Overall_quality	Notes
McClelland PH	2025	Melanoma (acral vs non-acral)	Retrospective cohort	Moderate	Moderate	Low	Low	Low	Moderate–High	Large dataset (n=442), comparative analysis
Mehta GU	2018	Melanoma (brain mets vs no brain mets)	Retrospective cohort	Moderate	High	Low	Low	Low	Moderate	Subgroup comparison; confounding high
Saint-Jean M	2018	Melanoma	Retrospective single-center	High	High	Low	Moderate	Moderate	Low–Moderate	Very small sample (n=10), exploratory

Supplementary Table 3: Risk of bias assessment of non-randomized single-arm studies

Study	Year	Tumor_type	Study_type	Selection_bias	Outcome_assessment	Follow_up	Reporting	Overall_quality	Notes
Baars JW	1992	Melanoma	Single-arm	High	Moderate	Poor	Moderate	Low	Very small pilot (n=4)
Goedegebuure PS	1995	Melanoma/RCC	Single-arm	High	Moderate	Moderate	Moderate	Low	Mixed tumor types
Dudley ME	2005	Melanoma	Single-arm	Moderate	Low	Adequate	Low	Moderate	Standard ACT protocol
Rosenberg SA	2011	Melanoma	Single-arm	Moderate	Low	Adequate	Low	Moderate	Large cohort, strong outcomes
Radvanyi L	2012	Melanoma	Single-arm	Moderate	Low	Adequate	Low	Moderate	Biomarker-driven study
Pilon-Thomas S	2012	Melanoma	Single-arm	Moderate	Moderate	Moderate	Moderate	Moderate	Small treated population
Ellebaek E	2012	Melanoma	Single-arm	Moderate	Low	Moderate	Low	Moderate	Low-dose IL-2 variation
Besser MJ	2013	Melanoma	Single-arm	Moderate	Low	Adequate	Low	Moderate	ITT vs treated variability
EU-CTR trial	2020	Melanoma	Single-arm	Moderate	Moderate	Moderate	Moderate	Moderate	Registry-based data
Khammari A	2015	Melanoma	Single-arm	Moderate	Moderate	Moderate	Moderate	Moderate	Combination (TG1042)
Jiang SS	2015	HCC	Single-arm	Moderate	Moderate	Moderate	Moderate	Moderate	Non-melanoma, post-surgery
Andersen R	2016	Melanoma	Single-arm	Moderate	Low	Adequate	Low	Moderate	Optimized IL-2 regimen
Chandran SS	2017	Uveal melanoma	Single-arm	Moderate	Low	Moderate	Low	Moderate	Rare tumor type
Forget MA	2018	Melanoma	Single-arm	Moderate	Low	Adequate	Low	Moderate	Large translational dataset
Nguyen LT	2019	Melanoma	Single-arm	Moderate	Low	Moderate	Low	Moderate	Low-dose IL-2
NCT01701674	2025	Melanoma	Single-arm	Moderate	Moderate	Moderate	Moderate	Moderate	Trial registry results
van den Berg JH	2020	Melanoma	Single-arm	Moderate	Low	Adequate	Low	Moderate	Long-term follow-up

Hirai I	2021	Melanoma	Single-arm	High	Moderate	Poor	Moderate	Low	Very small (n≈3)
Sarnaik A	2021	Melanoma	Single-arm	Moderate	Low	Adequate	Low	Moderate	Lifileucel study
Chesney J	2022	Melanoma	Single-arm	Moderate	Low	Adequate	Low	Moderate	Large multicenter dataset
König D	2024	Melanoma	Single-arm	Moderate	Low	Moderate	Low	Moderate	Small phase I combination with nivolumab; 11 enrolled, 9 treated
EU-CTR 2015-000530-30	2018	Ovarian cancer	Single-arm	High	Moderate	Moderate	Moderate	Low	Registry results; 11 harvested, 6 treated; feasibility-focused, small sample
Kverneland AH	2020	Ovarian cancer	Single-arm	Moderate	Moderate	Moderate	Low	Moderate	Combination ACT + checkpoint inhibitors; 7 recruited, 6 treated; small cohort
Amaria R	2024	CRC/PDAC/Ovarian	Single-arm	Moderate	Low	Adequate	Low	Moderate	Multicohort phase II; 17 harvested, 16 treated; objective endpoints clearly defined
NCT03083873	2023	HNSCC	Single-arm	Moderate	Moderate	Adequate	Moderate	Moderate	Registry-based multi-cohort LN-145/LN-145-S1 study; non-randomized, open-label
Sherif A	2010	Urothelial bladder cancer	Single-arm	High	Moderate	Poor	Moderate	Low	Feasibility/preliminary study; 12 included, only 6 infused due to technical failures
Wang C	2022	Osteosarcoma	Single-arm	Moderate	Moderate	Poor	Moderate	Low–Moderate	Exploratory single-arm study; 12 enrolled, 11 treated;

									short follow-up (1–13 months)

Supplementary Table 4: Primary efficacy outcomes of included studies

· Author<br>· Country<br>· Year	Clinical efficiency outcomes		Outcome observed	Response criteria	Median Follow-up	Duration of response	Time to respond
Melanoma/Metastatic melanoma							
**RCTs**							
Dréno B[14]<br><br>France<br><br>2002	Objective response rate		NR	NR	NR	NR	NR
	Complete response rate		NR				
	Partial response rate		NR				
	Disease control rate		NR				
	Progression-free survival	Months	NR				
		HR	NR				
	Overall survival	Months	Median OS: TIL + IL-2: 29.5 months, IL-2 only: 20.0 months, Difference: Not significant (P = 0.63)				
		HR	NR				
Khammari A[12]<br><br>France<br><br>2020	Objective response rate		NR	NR	5 Years	NR	NR
	Complete response rate		NR				
	Partial response rate		NR				
	Disease control rate		NR				
	Progression-free survival	Months	NR				
		HR	NR				
	Overall survival	Months	5-year OS (PP population):<br>TIL+IL-2: 70.8%<br>Control: 52.2%				

		HR	0.503 (95% CI 0.195–1.30), p = 0.148				
Rohaan MW [13]<br><br>Multinational<br><br>2022	Objective response rate		TIL: 49% (41/84)<br>Ipilimumab: 21% (18/84)	RECIST version 1.1 (primary)	~24 months (estimated; exact NR)	NR	NR
	Complete response rate		TIL: 20% (17/84)<br>Ipilimumab: 7% (6/84)				
	Partial response rate		TIL: 29% (24/84)<br>Ipilimumab: 14% (12/84)				
	Disease control rate		TIL: 68% (57/84) Ipilimumab: 39% (33/84)				
	Progression free survival	Months	TIL: 7.2 months<br>Ipilimumab: 3.1 months				
		HR	0.50 (95% CI 0.35–0.72; p<0.001)				
	Overall survival	Months	TIL: 25.8 months<br>Ipilimumab: 18.9 months				
		HR	0.83 (95% CI 0.54–1.27)				
non-RCTs (Single-arm studies)							
Baars JW[22]<br><br>Netherlands<br><br>1992	Objective response rate		0	WHO Criteria	NR	2.5-4 months (SD only)	NR
	Complete response rate		0				
	Partial response rate		0				
	Disease control rate		0.5				
	Progression-free survival	Months	NR				
		HR	NR				
	Overall survival	Months	NR				
		HR	NR				
P S Goedegebuure	Objective response rate		Melanoma: 19% (CR only); RCC: 0% ORR	WHO Criteria	NR	CRs durable: 15-33+months	NR
	Complete response rate		Melanoma: 19% (3/16 assessable)				

USA<br><br>1995	Partial response rate		0				
	Disease control rate		NR				
	Progression-free survival	Months	NR				
		HR	NR				
	Overall survival	Months	NR				
		HR	NR				
Dudley ME[23]<br><br>USA<br><br>2005	Objective response rate		(ORR), 51% (18/35)	WHO-style bidimensional criteria	NR	Mean 11.5 ± 2.2 months	NR
	Complete response rate		9% (3/35)				
	Partial response rate		43% (15/35)				
	Disease control rate		NR				
	Progression-free survival	Months	NR				
		HR	NR				
	Overall survival	Months	NR				
		HR	NR				
Rosenberg SA[24]<br><br>USA<br><br>2011	Objective response rate		56% (52/93)	RECIST	62 months	CR ongoing 37–82+ months	NR
	Complete response rate		22% (20/93)				
	Partial response rate		34% (32/93)				
	Disease control rate		NR				
	Progression-free survival	Months	NR				
		HR	NR				
	Overall survival	Months	5-year OS (all patients): 29%<br>3-year OS (CR patients): 100%<br>5-year OS (CR patients): 93%				
		HR	NR				

Radvanyi LG[25]<br><br>USA<br><br>2012	Objective response rate		Objective response rate (ORR): 48.4% (15/31) by irRC	IrRC (primary), RECIST 1.1 (secondary)	NR	NR	NRS
	Complete response rate		Complete response rate: 6.5% (2/31)				
	Partial response rate		41.9% (13/31)				
	Disease control rate		NR				
	Progression-free survival	Months	7.6 months (95% CI 4.1-22.2); 6-month PFS: 57%				
		HR	NR				
	Overall survival	Months	NR				
		HR	NR				
Pilon-Thomas S[26]<br><br>USA<br><br>2012	Objective response rate		Treated patients: 38% (5/13)<br>Intention-to-treat: 26% (5/19)	RECIST 1.1	10 months	CR: up to 16+ months<br>PR: up to 19+ months<br>SD: up to 24+ months	NR
	Complete response rate		2/13 (15%)				
	Partial response rate		3/13 (23%)				
	Disease control rate		(CR+PR+SD): 69% (9/13)				
	Progression-free survival	Months	NR				
		HR	NR				
	Overall survival	Months	NR				
		HR	NR				
Ellebaek E[27]<br><br>Denmark<br><br>2012	Objective response rate		33% (2/6)	RECIST 1.0	NR	Ongoing CRs at 10+ and 30+ months	First assessment at 8 weeks
	Complete response rate		33% (2/6)				
	Partial response rate		0				
	Disease control rate		67% (CR + SD = 4/6)				
	Progression-free survival	Months	Median TTP 8.2 months (range 2–30)				

		HR	NR				
	Overall survival	Months	Median OS 12 months (range 5–30)				
		HR	NR				
Besser MJ[28]<br><br>Israel<br><br>2013	Objective response rate		29% (23/80) ITT;<br>40% (23/57) treated	RECIST 1.1	28 months	CRs ongoing at 13-52 months	NR
	Complete response rate		6% ITT; 9% treated<br>(5 patients)				
	Partial response rate		23% ITT; 32% treated				
	Disease control rate		65% treated<br>(CR + PR + SD)				
	Progression-free survival	Months	4.0 months (treated)				
		HR	NR				
	Overall survival	Months	15.2 months (treated)				
		HR	NR				
EUCTR_2014-001419-38[40]<br>Denmark	Objective response rate		9/12 (75%)	RECIST 1.1	NR	NR	NR
	Complete response rate		CR: 1, 0.083				
	Partial response rate		PR: 8, 0.667				
	Disease control rate		(CR+PR+SD): 100%				
	Progression-free survival	Months	4.8 months<br>(range 2.5–36.1)				
		HR	NR				
	Overall survival	Months	28.8 months<br>(range 5.2–39.9)				
		HR	NR				
	Objective response rate		38.5% (5/13)	RECIST 1.1	30 months		NR

Khammari A[29]<br>France<br>2015	Complete response rate		23% (3/13)			CRs: 15, 17+, and 35+ months	
	Partial response rate		15% (2/13)				
	Disease control rate		46% (CR + PR + SD)				
	Progression-free survival	Months	NR				
		HR	NR				
	Overall survival	Months	21.1 months (95% CI 12.1-NR)				
		HR	NR				
	Complete response rate		23% (3/13)				
	Partial response rate		15% (2/13)				
	Disease control rate		46% (CR + PR + SD)				
	Progression-free survival	Months	NR				
		HR	NR				
	Overall survival	Months	21.1 months<br>(95% CI 12.1–NR)				
		HR	NR				
AndersenR[31]<br>Denmark<br>2016	Objective response rate		42% (10/24)	RECIST v 1.0	28.1 months	CRs and some PRs ongoing upto 45+ months	NR
	Complete response rate		13% (3/24)				
	Partial response rate		29% (7/24)				
	Disease control rate		(CR+PR+SD): 88%				
	Progression-free survival	Months	3.9 months				
		HR	NR				
	Overall survival	Months	21.8 months				
		HR	NR				

Chandran SS[32]<br><br>USA<br><br>2017	Objective response rate		35% (7/20 evaluable; 95% CI 16–59)	RECIST v 1.0	7.3 months (IQR 4.4–17.0)	PR: 3–9 months<br>CR: ongoing ≥21 months	~1 month (median)
	Complete response rate		5% (1/20)				
	Partial response rate		30% (6/20)				
	Disease control rate		NR				
	Progression-free survival	Months	NR				
		HR	NR				
	Overall survival	Months	NR				
		HR	NR				
Forget MA[33]<br><br>USA<br><br>2018	Objective response rate		Overall: 42% (31/74), Subgroup ORR: Checkpoint-naïve: 47%, Anti-CTLA-4 exposed: 38%, Anti-CTLA-4 + PD-1 exposed: 33%	Immune-related response criteria (irRC)	74 months	Checkpoint-naïve: 69.1 months<br>Anti-CTLA-4 exposed: 4.8 months	NR
	Complete response rate		11% (8/74)				
	Partial response rate		31% (23/74)				
	Disease control rate		NR				
	Progression-free survival	Months	4.0 months (95% CI 2.9–5.8)				
		HR	NR				
	Overall survival	Months	17.3 months (95% CI 11.6–37.8)				
		HR	NR				
Nguyen LT[34]<br><br>Canada	Objective response rate		RECIST: 2 PR + 1 PRu → 25% (3/12)<br>irRC: 3 confirmed PR (25%)	RECIST v1.1; irRC	NR	Responses not durable; no CRs	At first radiographic assessment
	Complete response rate		0				
	Partial response rate		16.7% confirmed				

2019	Disease control rate		75% (9/12)				
	Progression-free survival	Months	5.1 months (95% CI 1.2–6.4)				
		HR	NR				
	Overall survival	Months	6.2 months				
		HR	NR				
NCT01701674[41]<br><br>USA	Objective response rate		38.50%	RECIST v1.1	NR	NR	NR
	Complete response rate		NR				
	Partial response rate		NR				
	Disease control rate		NR				
	Progression-free survival	Months	7.4 months (range 1.5-42)				
		HR	NR				
	Overall survival	Months	NR				
		HR	NR				
Van den Berg JH[35]<br>Netherlands<br><br>2020	Objective response rate		(ORR): 50% (5/10)	RECIST	>7 years (long-term follow-up reported)	CRs durable (>84–108 months)	~1–3 months
	Complete response rate		20% (2/10)				
	Partial response rate		30% (3/10)				
	Disease control rate		0.6				
	Progression-free survival	Months	NR				
		HR	NR				
	Overall survival	Months	Two CRs ongoing at >7 years and >9 years				
		HR	NR				
Hirai I[36]	Objective response rate		33.3%<br>(1 PR / 3 treated)	RECIST v1.1	NR	Short-lived PR (~2 months)	~4-6 week post infusion

Japan<br><br>2021	Complete response rate		0				
	Partial response rate		0.333				
	Disease control rate		66.7% (PR + SD)				
	Progression-free survival	Months	Patient-level PFS: 3.0-9.3 months				
		HR	NR				
	Overall survival	Months	Patient level OS: 7.0-13.4 months				
		HR	NR				
Sarnaik AA[37]<br><br>Multinational (USA, Europe)<br><br>2021	Objective response rate		36% (24/66; 95% CI 25–49)	RECIST v1.1	18.7 months	NR	Median 1.4 months
	Complete response rate		3% (2/66)				
	Partial response rate		33% (22/66)				
	Disease control rate		(CR + PR + SD): 80%				
	Progression-free survival	Months	NR				
		HR	NR				
	Overall survival	Months	17.4 months (95% CI 11.0-NR)				
		HR	NR				
Chesney J[38]<br><br>Multinational (USA, Germany, UK)<br><br>2022	Objective response rate		31.4%<br>(95% CI 24.1–39.4)	RECIST v1.1	27.6 months	Not reached	Median 1.5 months
	Complete response rate		5.2% (8/153)				
	Partial response rate		26.1% (40/153)				
	Disease control rate		0.778				
	Progression-free survival	Months	4.1 months<br>(95% CI 2.8–4.4)				
		HR	NR				

	Overall survival	Months	13.9 months (95% CI 10.6–17.8)				
		HR	NR				
König D[39] Switzerland 2024	Objective response rate		22% (2/9)	RECIST v1.1	NR	One patient >12 months	First imaging 1 month post TIL
	Complete response rate		0				
	Partial response rate		0.22				
	Disease control rate		56% (PR + SD = 5/9)				
	Progression-free survival	Months	2.2 months				
		HR	NR				
	Overall survival	Months	7.2 months				
		HR	NR				
Retrospective studies							
Saint-Jean [21] 2018 France	Objective response rate		20% (CR 1, PR 1)	RECIST 1.1	NR	PR lasted 8 months; SD up to 17 months	NR
	Complete response rate		0.1				
	Partial response rate		0.1				
	Disease control rate		Disease control rate 40% (CR + PR + SD)				
	Progression-free survival	Months	NR				
		HR	NR				
	Overall survival	Months	NR				
		HR	NR				
Mehta GU[19] USA 2018	Objective response rate		No brain mets (n=111) 49% Treated brain mets (n=15) 33% Untreated brain mets (n=18) 33%; Intracranial ORR: 28% (5/18)	RECIST v1.0	Median follow-up: 1.1 years (0.1–13.5)	Durable CRs reported (up to 9 years)	NR

			CR: 4 (22%)<br>PR: 1 (6%)				
	Complete response rate		No brain mets (n=111) 21%<br>Treated brain mets (n=15) 7%<br>Untreated brain mets (n=18) 0%				
	Partial response rate		No brain mets (n=111) 28%<br>Treated brain mets (n=15) 26%<br>Untreated brain mets (n=18) 33%				
	Disease control rate		NR				
	Progression-free survival	Months	Significantly longer in patients without brain mets vs untreated brain mets (p = 0.004)				
		HR	NR				
	Overall survival	Months	Significantly longer in patients without brain mets vs untreated brain mets (p = 0.02)				
		HR	NR				
McClelland PH[20]<br><br>USA<br><br>2025	Objective response rate		43% (95% CI 27–61)	RECIST 1.0	NR	Median 9 months	NR
	Complete response rate		0.03				
	Partial response rate		0.4				
	Disease control rate		NR				
	Progression-free survival	Months	3.5 months<br>(95% CI 2.3–4.8)				
		HR	NR				
	Overall survival	Months	13 months<br>(95% CI 7–37)				
		HR	NR				
**Carcinoma Ovary**							
non-RCTs (Two-arm studies)							

Fujita K[18]<br><br>Japan<br><br>1995	Objective response rate		NR	NR	TIL group: 36 months (range 23–44)<br>Control group: 33 months (range 14–48)	NR	NR
	Complete response rate		NR				
	Partial response rate		NR				
	Disease control rate		NR				
	Progression-free survival	Months	3-year Disease-Free Survival:<br>TIL group: 82.1%<br>Control group: 54.5% ($P < 0.05$)				
		HR	NR				
	Overall survival	Months	NR				
		HR	NR				
non-RCTs (Single-arm studies)							
EUCTR_2015-000530-30[40]<br>Denmark	Objective response rate		NR	NR	NR	NR	NR
	Complete response rate		NR				
	Partial response rate		NR				
	Disease control rate		NR				
	Progression-free survival	Months	6 months				
		HR	NR				
	Overall survival	Months	10 months				
		HR	NR				
Kverneland[42]<br><br>Denmark	Objective response rate		1 PR / 6 patients = 16.7%	RECIST 1.1	NR	NR	NR
	Complete response rate		0				
	Partial response rate		1 patient				
	Disease control rate		(PR + SD) = 6/6 = 100%				

2020	Progression-free survival	Progression-free survival	Median: 86 days (~2.8 months)				
		HR	NR				
	Overall survival	Overall survival	Median: 214 days (~7 months)				
		HR	NR				
**Carcinoma Colon**							
non-RCTs (Two-arm studies)							
Gardini A[17]<br>Italy<br>2004	Objective response rate		NR	Not specified	42 months (TIL group) vs 36 months (control)	NR	NR
	Complete response rate		NR				
	Partial response rate		NR				
	Disease control rate		NR				
	Progression-free survival	Months	NR				
		HR	NR				
	Overall survival	Months	1-year: 85% (TIL) vs 90% (control)<br>3-year: 55% vs 50%<br>5-year: 25% vs 38%<br>(OS: P=0.7)				
		HR	NR				
non-RCTs (Single-arm studies)							
Amaria R[43]<br>USA<br>2024	Objective response rate		(ORR): 0% (no CR or PR)	RECIST v1.1	18.9 months (range 2.2–44.8)	One PDAC patient with prolonged SD 17 months	NR
	Complete response rate		0				
	Partial response rate		0				
	Disease control rate		62.5%<br>(95% CI 35.4–84.8%)				

	Progression-free survival	Months	2.53 months (95% CI 1.54–4.11)				
		HR	NR				
	Overall survival	Months	18.86 months (95% CI 4.86–NR)				
		HR	NR				
**NSCLC**							
RCT							
Ratto GB[15] Italy 1996	Objective response rate		NR	NR	6–40 months	NR	NR
	Complete response rate		NR				
	Partial response rate		NR				
	Disease control rate		NR				
	Progression-free survival	Months	NR				
		HR	NR				
	Overall survival	Months	NR				
		HR	Median OS: Control: 14.1 months/ AI arm: 22.4 months				
**Carcinoma Nasopharynx**							
RCT							
Liang YJ[16] China 2023	Objective response rate		97.4% in CCRT+TIL; 94.90% in CCRT alone	NR	62.3 months (IQR 56.3-76.7)	NR	NR
	Complete response rate		88.5% in CCRT+TIL; 83.30% in CCRT alone				
	Partial response rate		9% in CCRT+TIL; 11.5% CCRT alone				
	Disease control rate		NR				

	Progression-free survival	Months	NR				
		HR	HR 1.08<br>(95% CI 0.62–1.89)				
	Overall survival	Months	NR				
		HR	HR 1.15<br>(95% CI 0.57–2.29)				
**Head and Neck SCC**							
non-RCTs (Single-arm studies)							
NCT03083873[44]<br><br>USA	Objective response rate		Cohort 1: 37.5% (95% CI 8.5–75.5)<br>Cohort 2: 5.9% (95% CI 0.1–28.7)<br>Cohort 3: 0% (95% CI 0–20.6)	RECIST v1.1	NR	Median 3.1 months in Cohort 1 (others NR)	NR
	Complete response rate		NR				
	Partial response rate		NR				
	Disease control rate		Cohort 1: 87.5%<br>Cohort 2: 70.6%<br>Cohort 3: 75.0%				
	Progression-free survival	Months	Cohort 1: 2.8 (0.9–3.9)<br>Cohort 2: 1.9 (1.6–2.8)<br>Cohort 3: 2.8 (1.8–3.1)				
		HR	NR				
	Overall survival	Months	NR				
		HR	NR				
**Hepatocellular carcinoma**							
non-RCTs (Single-arm studies)							
Jiang SS[30]	Objective response rate		NR	WHO criteria and/or RECIST	14 months (range 12–16)	NR	NR
	Complete response rate		No evidence of disease in 12/15 (80%)				

China	Partial response rate		NR				
	Disease control rate		80% NED				
2015	Progression-free survival	Months	recurrence in 3 patients at 105–261 days				
		HR	NR				
	Overall survival	Months	NR				
		HR	NR				
**Osteosarcoma**							
non-RCTs (Single-arm studies)							
Wang C[45]	Objective response rate		0				
	Complete response rate		0				
China	Partial response rate		0				
	Disease control rate		81.8% (9/11 SD)				
2022	Progression-free survival	Months	4.1 months				
		HR	NR				
	Overall survival	Months	7.6 months				
		HR	NR				

NR, not reported; ORR, objective response rate; CR, complete response; PR, partial response; SD, stable disease; DCR, disease control rate; PFS, progression-free survival; OS, overall survival; DFS, disease-free survival; DOR, duration of response; TTP, time to progression; HR, hazard ratio; CI, confidence interval; IQR, interquartile range; RECIST, Response Evaluation Criteria in Solid Tumors; irRC, immune-related response criteria; WHO, World Health Organization criteria; IRC, independent review committee; ITT, intention-to-treat; PP, per-protocol.

Supplementary Table 5: Secondary outcomes: safety, manufacturing feasibility, and vein-to-vein time

· Author<br>· Country<br>· Year	Safety outcomes	Outcome observed	Manufacturing success rate	Vein to vein time
		**Melanoma/ metastatic melanoma**		
RCTs				
Dréno B[14]<br>France<br>2002	Grade ≥3 toxicity	No severe toxicities reported	100% (88/88 expansions successful)	NR
	Cytokine-related toxicity	Mild IL-2 related		
	Autoimmune toxicity	NR		
	Treatment-related deaths	0		
	Neuro toxicity	NR		
	Hematological toxicity	None ≥ grade 3		
Khammari A[12]<br>France<br>2020	Grade ≥3 toxicity	Rare	NR	NR
	Cytokine-related toxicity	Common but mostly mild (IL-2 related)		
	Autoimmune toxicity	Vitiligo reported (2 patients per arm)		
	Treatment-related deaths	0		
	Neuro toxicity	NR		
	Hematological toxicity	Mild, transient		
Rohaan MW[13]<br>Multinational<br>2022	Grade ≥3 toxicity	~100% (TIL) vs 57% (control)	80/84 (95.2%) received TIL, but only 1 patient non-receipt of TIL was manufacturing failure.	~4–6 weeks (~5 weeks)
	Cytokine-related toxicity	Present (high-dose IL-2–related; fever, hypotension)		
	Autoimmune toxicity	Present (vitiligo, uveitis)		
	Treatment-related deaths	NR		
	Neuro toxicity	NR		

	Hematological toxicity	Severe myelosuppression (cyclophosphamide + fludarabine)		
non-RCTs (Single-arm studies)				
Baars JW[22] Netherlands 1992	Grade ≥3 toxicity	Yes (frequent)	4/4 (100%)	25–40 days
	Cytokine-related toxicity	Fever, hypotension, neuropsychiatric effects		
	Autoimmune toxicity	NR		
	Treatment-related deaths	1 death (pseudomonas septicaemia during therapy)		
	Neuro toxicity	Confusion, disorientation, psychosis		
	Hematological toxicity	Anemia (transfusions required)<br>Thrombocytopenia ($<50 \times 10^9$/L)<br>Febrile neutropenia		
P S Goedegebuure[42] USA 1995	Grade ≥3 toxicity	Yes	100% (26/26 expanded to target dose)	~6–8 weeks
	Cytokine-related toxicity	Yes (IL-2 related)		
	Autoimmune toxicity	NR		
	Treatment-related deaths	NO		
	Neuro toxicity	Confusion, hallucinations (reversible)		
	Hematological toxicity	Thrombocytopenia, cytopenias		
Dudley ME[23] USA 2005	Grade ≥3 toxicity	Common (chemotherapy + IL-2 related)	NR	NR
	Cytokine-related toxicity	Yes (IL-2 related)		
	Autoimmune toxicity	Vitiligo 37%; uveitis 14%		
	Treatment-related deaths	No		
	Neuro toxicity	Cortical blindness 3% (fludarabine-related)		
	Hematological toxicity	Universal neutropenia, thrombocytopenia		
	Grade ≥3 toxicity	Common (lymphodepletion + IL-2 related)		NR

Rosenberg SA[24]<br><br>USA<br><br>2011	Cytokine-related toxicity	IL-2–associated (expected)	Viable TIL grown in 75–85% of resected tumors	
	Autoimmune toxicity	NR		
	Treatment-related deaths	1/93 (1.1%) – sepsis post-TBI		
	Neuro toxicity	NR		
	Hematological toxicity	Universal		
Radvanyi LG[25]<br><br>USA<br><br>2012	Grade ≥3 toxicity	Yes (mainly IL-2–related and lymphodepletion-related)	NR	NR
	Cytokine-related toxicity	Typical HD IL-2 toxicities (pulmonary, renal, hepatic, neurocognitive)		
	Autoimmune toxicity	NR		
	Treatment-related deaths	0		
	Neuro toxicity	Transient mental confusion during IL-2		
	Hematological toxicity	Universal transient neutropenia and lymphopenia		
Pilon-Thomas S[26]<br><br>USA<br><br>2012	Grade ≥3 toxicity	100% (expected IL-2 related)	TIL grown from 17/19 (89%) tumors	Median 47 days from surgery to infusion
	Cytokine-related toxicity	Severe IL-2 toxicities common but reversible		
	Autoimmune toxicity	Vitiligo<br>Uveitis (required steroids)		
	Treatment-related deaths	0		
	Neuro toxicity	Hearing loss (1 patient)		
	Hematological toxicity	Universal transient cytopenias<br>Transfusions required in 12 patients		
Ellebaek E[27]<br><br>Denmark	Grade ≥3 toxicity	Yes (chemotherapy-related)	NR	NR
	Cytokine-related toxicity	No grade 3–4 IL-2 toxicities		
	Autoimmune toxicity	Not reported		

2012	Treatment-related deaths	NR		
	Neuro toxicity	None reported		
	Hematological toxicity	Grade 4 leukopenia/neutropenia/lymphopenia		
Besser MJ[28]<br><br>Israel<br><br>2013	Grade ≥3 toxicity	Yes (≈93%)	72/80 (90%) culture success	NR
	Cytokine-related toxicity	Yes (IL-2 related)		
	Autoimmune toxicity	Rare (vitiligo 2%)		
	Treatment-related deaths	1 death (cardiac failure during conditioning)		
	Neuro toxicity	Confusion 7%		
	Hematological toxicity	Universal cytopenias (grade 3–4)		
EUCTR_2014-001419-38[40] Denmark	Grade ≥3 toxicity	Reported (35 grade 3–4 AEs)	12/13 successfully infused (~92%)	NR
	Cytokine-related toxicity	IL-2–associated toxicities present (not individually categorized as cytokine release syndrome)		
	Autoimmune toxicity	Uveitis (8.3%), Vitiligo (8.3%)		
	Treatment-related deaths	0		
	Neuro toxicity	Delirium (16.7%), Hallucinations (16.7%), Peripheral neuropathy (8.3%)		
	Hematological toxicity	Febrile neutropenia: 100%, Neutropenia, lymphopenia, thrombocytopenia: 100%		
Khammari A[29]<br><br>France<br><br>2015	Grade ≥3 toxicity	Yes (6 grade-3 AEs in 5 patients)	16/18 TILs expanded; 13/18 infused	NR
	Cytokine-related toxicity	Yes (IL-2 / IFN-γ related)		
	Autoimmune toxicity	None observed		
	Treatment-related deaths	NR		
	Neuro toxicity	None reported		

AndersenR[31]<br><br>Denmark<br><br>2016	Grade ≥3 toxicity	Common (hematologic, IL-2 related)	32/33 tumors successfully expanded (~97%)	NR
	Cytokine-related toxicity	Fever (88%), hypotension, fluid retention, GI & neurologic symptoms		
	Autoimmune toxicity	Vitiligo (n = 2), uveitis (n = 1), vasculitis (n = 1), pernicious anemia (n = 1)		
	Treatment-related deaths	Intratumoral hemorrhage in previously irradiated brain metastasis		
	Neuro toxicity	Delirium (n = 2)		
	Hematological toxicity	Febrile neutropenia 96%<br>Universal transient cytopenias		
Rosenberg SA[24]<br><br>USA<br><br>2011	Grade ≥3 toxicity	Common (lymphodepletion + IL-2 related)	Viable TIL grown in 75–85% of resected tumors	NR
	Cytokine-related toxicity	IL-2–associated (expected)		
	Autoimmune toxicity	NR		
	Treatment-related deaths	1/93 (1.1%) – sepsis post-TBI		
	Neuro toxicity	NR		
	Hematological toxicity	Universal		
Amaria R[43]<br><br>USA<br><br>2024	Grade ≥3 toxicity	87.5% (14/16)	16/17 harvested successfully treated (~94%)	NR
	Cytokine-related toxicity	Expected IL-2 related AEs		
	Autoimmune toxicity	NR		
	Treatment-related deaths	0 (grade 5 AEs unrelated)		
	Neuro toxicity	No		
	Hematological toxicity	Grade 4 thrombocytopenia: 75%<br>Grade 4 neutropenia: 25%<br>Febrile neutropenia present		
Chesney J[38]	Grade ≥3 toxicity	Very common	94.7% (177/187)	NR
	Cytokine-related toxicity	IL-2–associated (capillary leak, hypotension, fever)		

Multinational (USA, Germany, UK)<br><br>2022	Autoimmune toxicity	Grade 3/4 uveitis: 3 patients		
	Treatment-related deaths	4 grade-5 AEs (mostly LD/IL-2 related; 1 regimen-related)		
	Neuro toxicity	Rare (1 patient with ICANS/CRS)		
	Hematological toxicity	Thrombocytopenia (76.9%)<br>Anemia (50.0%)<br>Febrile neutropenia (41.7%)		
Pilon-Thomas S[26]<br><br>USA<br><br>2012	Grade ≥3 toxicity	100% (expected IL-2 related)	TIL grown from 17/19 (89%) tumors	Median 47 days from surgery to infusion
	Cytokine-related toxicity	Severe IL-2 toxicities common but reversible		
	Autoimmune toxicity	Vitiligo<br>Uveitis (required steroids)		
	Treatment-related deaths	0		
	Neuro toxicity	Hearing loss (1 patient)		
	Hematological toxicity	Universal transient cytopenias<br>Transfusions required in 12 patients		
NCT01585428<br><br>USA	Grade ≥3 toxicity	Common	NR	NR
	Cytokine-related toxicity	IL-2–associated toxicities (hypotension, fever, capillary leak)		
	Autoimmune toxicity	NR		
	Treatment-related deaths	0		
	Neuro toxicity	Confusion, hallucinations, syncope (≤18%)		
	Hematological toxicity	Neutropenia, lymphopenia, thrombocytopenia: >90%<br>Febrile neutropenia: ~33%		
NCT01701674[41]	Grade ≥3 toxicity	Yes	100% TIL growth from resected tumors	Mean 51 days (range 39–77)
	Cytokine-related toxicity	IL-2-related hypotension, hypoxemia, electrolyte disturbances		

USA	Autoimmune toxicity	Hypothyroidism: 3, Hepatitis: 2, Uveitis: 1, Colitis: 1		
	Treatment-related deaths	0		
	Neuro toxicity	Presyncope/syncope (Grade 3): 1		
	Hematological toxicity	Febrile neutropenia: 10/12, Leukopenia, thrombocytopenia: common		
Sarnaik AA[37]<br><br>Multinational (USA, Europe)<br><br>2021	Grade ≥3 toxicity	0.97	TIL successfully manufactured in 96% of harvested patients	NR (manufacturing time reported; full vein-to-vein not specified)
	Cytokine-related toxicity	IL-2–related hypotension, fever, capillary leak		
	Autoimmune toxicity	No recurrence of prior ICI-related irAEs reported		
	Treatment-related deaths	One possibly TIL-related (intra-abdominal hemorrhage)		
	Neuro toxicity	Confusion, hallucinations (≤ ~10%)		
	Hematological toxicity	Febrile neutropenia 55%<br>Thrombocytopenia 82%<br>Anemia 56%		
AndersenR[31]<br><br>Denmark<br><br>2016	Grade ≥3 toxicity	Common (hematologic, IL-2 related)	32/33 tumors successfully expanded (~97%)	NR
	Cytokine-related toxicity	Fever (88%), hypotension, fluid retention, GI & neurologic symptoms		
	Autoimmune toxicity	Vitiligo (n = 2), uveitis (n = 1), vasculitis (n = 1), pernicious anemia (n = 1)		
	Treatment-related deaths	Intratumoral hemorrhage in previously irradiated brain metastasis		
	Neuro toxicity	Delirium (n = 2)		
	Hematological toxicity	Febrile neutropenia 96%<br>Universal transient cytopenias		
Chandran SS[32]	Grade ≥3 toxicity	100% (expected hematologic)	96% (26/27)	NR
	Cytokine-related toxicity	IL-2-associated (mostly reversible)		

USA<br><br>2017	Autoimmune toxicity	Vitiligo (5%)		
	Treatment-related deaths	1 (5%) – sepsis-related		
	Neuro toxicity	Delayed grade 4 neurologic toxicity (5%)		
	Hematological toxicity	Lymphopenia, neutropenia, thrombocytopenia: 100%<br>Grade 3 anemia: 67%		
Forget MA[33]<br><br>USA<br><br>2018	Grade ≥3 toxicity	Yes (expected)	~10% of harvested tumors proceeded to REP	NR
	Cytokine-related toxicity	IL-2 associated (capillary leak, hyperbilirubinemia)		
	Autoimmune toxicity	NR		
	Treatment-related deaths	NR		
	Neuro toxicity	NR		
	Hematological toxicity	Neutropenia, anemia, thrombocytopenia, febrile neutropenia common		
Nguyen LT[34]<br><br>Canada<br><br>2019	Grade ≥3 toxicity	100% (expected from lymphodepletion)	12/12 (100%)	NR
	Cytokine-related toxicity	Mostly grade 1–2 IL-2 toxicities<br>Capillary leak, edema, hypotension (manageable, non-ICU)		
	Autoimmune toxicity	Vitiligo: 2 patients		
	Treatment-related deaths	0		
	Neuro toxicity	Hallucinations, mental status changes (low frequency)		
	Hematological toxicity	Universal grade 3–4 cytopenias<br>Febrile neutropenia common		
NCT01701674[41]	Grade ≥3 toxicity	Yes	100% TIL growth from resected tumors	Mean 51 days (range 39–77)
	Cytokine-related toxicity	IL-2-related hypotension, hypoxemia, electrolyte disturbances		

USA	Autoimmune toxicity	Hypothyroidism: 3, Hepatitis: 2, Uveitis: 1, Colitis: 1		
	Treatment-related deaths	0		
	Neuro toxicity	Presyncope/syncope (Grade 3): 1		
	Hematological toxicity	Febrile neutropenia: 10/12, Leukopenia, thrombocytopenia: common		
van den Berg JH[35] Netherlands 2020	Grade ≥3 toxicity	Yes (100%)	13/13 (100%)	~5–6 weeks
	Cytokine-related toxicity	Febrile neutropenia (100%)<br>Hypotension, dyspnea, chills, tachycardia		
	Autoimmune toxicity	1 patient (uveitis / VKH-like syndrome)		
	Treatment-related deaths	0		
	Neuro toxicity	Transient confusion (IL-2 related)		
	Hematological toxicity	Grade 4 neutropenia (100%)<br>Grade 3–4 thrombocytopenia (70%)<br>Grade 3–4 anemia (30%)		
Hirai I[36] Japan 2021	Grade ≥3 toxicity	Yes	3/4 (75%)	NR
	Cytokine-related toxicity	Yes (fever, nausea, diarrhea, metabolic disturbances)		
	Autoimmune toxicity	No		
	Treatment-related deaths	0		
	Neuro toxicity	None reported		
	Hematological toxicity	Grade 4 leukopenia/neutropenia (reversible)		
Sarnaik AA[37] Multinational (USA, Europe)	Grade ≥3 toxicity	0.97	TIL successfully manufactured in 96% of harvested patients	NR
	Cytokine-related toxicity	IL-2–related hypotension, fever, capillary leak		
	Autoimmune toxicity	No recurrence of prior ICI-related irAEs reported		

2021	Treatment-related deaths	One possibly TIL-related (intra-abdominal hemorrhage)		
	Neuro toxicity	Confusion, hallucinations (≤ ~10%)		
	Hematological toxicity	Febrile neutropenia 55%<br>Thrombocytopenia 82%<br>Anemia 56%		
Chesney J[38]<br><br>Multinational (USA, Germany, UK)<br><br>2022	Grade ≥3 toxicity	Very common	94.7% (177/187)	NR
	Cytokine-related toxicity	IL-2–associated (capillary leak, hypotension, fever)		
	Autoimmune toxicity	Grade 3/4 uveitis: 3 patients		
	Treatment-related deaths	4 grade-5 AEs (mostly LD/IL-2 related; 1 regimen-related)		
	Neuro toxicity	Rare (1 patient with ICANS/CRS)		
	Hematological toxicity	Thrombocytopenia (76.9%)<br>Anemia (50.0%)<br>Febrile neutropenia (41.7%)		
König D[39]<br><br>Switzerland<br><br>2024	Grade ≥3 toxicity	100% (hematologic)	NR	NR
	Cytokine-related toxicity	Yes (CRS 56%; 1 grade 4)		
	Autoimmune toxicity	Yes (grade 1 thyroiditis, adrenal insufficiency, vitiligo)		
	Treatment-related deaths	No		
	Neuro toxicity	Yes (part of grade 4 CRS, 1 patient)		
	Hematological toxicity	Yes (grade 4 neutropenia, lymphopenia common)		
Retrospective studies				
Saint-Jean[21]<br><br>2018	Grade ≥3 toxicity	1 patient (grade 3 nausea/vomiting)	100% (10/10)	~5 weeks
	Cytokine-related toxicity	Yes (IL-2 related)		
	Autoimmune toxicity	No		

France	Treatment-related deaths	No		
	Neuro toxicity	No		
	Hematological toxicity	Mild thrombocytopenia (grade 1–2)		
Mehta GU[19]<br>USA<br>2018	Grade ≥3 toxicity	Yes (expected LD + IL-2 related)	NR	NR
	Cytokine-related toxicity	IL-2 related toxicities common		
	Autoimmune toxicity	NR		
	Treatment-related deaths	0		
	Neuro toxicity	Rare; one symptomatic intracerebral hemorrhage (untreated brain mets cohort)		
	Hematological toxicity	Universal cytopenias; platelet support required in brain mets patients		
McClelland PH[20]<br>USA<br>2025	Grade ≥3 toxicity	0.967	NR	NR
	Cytokine-related toxicity	Yes (IL-2 related)		
	Autoimmune toxicity	No		
	Treatment-related deaths	6.7% (grade 5 AEs)		
	Neuro toxicity	No		
	Hematological toxicity	Common (per lymphodepletion)		
**Carcinoma Ovary**				
non-RCTs (Two-arm studies)				
Fujita K[18]<br>Japan<br>1995	Grade ≥3 toxicity	No severe toxicities reported		NR
	Cytokine-related toxicity	NR		
	Autoimmune toxicity	None observed		
	Treatment-related deaths	0		
	Neuro toxicity	NR		

	Hematological toxicity	NR		
**non-RCTs (Single-arm studies)**				
EUCTR_2015-000530-30 Denmark	Grade ≥3 toxicity	Yes (expected lymphodepletion/IL-2 related)	Tissue collected from 11 patients; 6 successfully treated	NR
	Cytokine-related toxicity	Fever, electrolyte disturbances, febrile neutropenia		
	Autoimmune toxicity	NR		
	Treatment-related deaths	0		
	Neuro toxicity	Confusional state (16.7%), Hallucinations (16.7%)		
	Hematological toxicity	Febrile neutropenia: 100%, Cytopenias: 100%		
**Carcinoma Colon**				
non-RCTs (Two-arm studies)				
Gardini A[17]<br><br>Italy<br><br>2004	Grade ≥3 toxicity	3/14 (21%)	14/25 (56%) received therapy Failure due to: Poor TIL expansion and Consent withdrawal 3/4 (75%)	40–71 days (median ~52 days)
	Cytokine-related toxicity	Fever, Hypotension, Oliguria, Tachycardia		
	Autoimmune toxicity	NR		
	Treatment-related deaths	0		
	Neuro toxicity	1 patient (epileptic fit)		
	Hematological toxicity	NR		
non-RCTs (Single-arm studies)				
Amaria R[43]<br><br>USA	Grade ≥3 toxicity	87.5% (14/16)	16/17 harvested successfully treated (~94%)	NR
	Cytokine-related toxicity	Expected IL-2 related AEs		
	Autoimmune toxicity	NR		
	Treatment-related deaths	0 (grade 5 AEs unrelated)		

2024	Neuro toxicity	No		
	Hematological toxicity	Grade 4 thrombocytopenia: 75%<br>Grade 4 neutropenia: 25%<br>Febrile neutropenia present		
**NSCLC**				
RCT				
Ratto GB[15]<br><br>Italy<br><br>1996	Grade ≥3 toxicity	No grade IV events	~86% (113/131 cultures successful)	~6–8 weeks post-surgery
	Cytokine-related toxicity	Yes (fever, chills, malaise, nausea)		
	Autoimmune toxicity	NO		
	Treatment-related deaths	NR		
	Neuro toxicity	NR		
	Hematological toxicity	NO		
**Carcinoma Nasopharynx**				
RCT				
Liang YJ[16]<br><br>China<br><br>2023	Grade ≥3 toxicity	Yes (but attributable to CCRT; TIL caused no grade 3–4 AEs)	83.3% (65/78)	Not reported; only CCRT completion→infusion median 3 days (IQR 1–5)
	Cytokine-related toxicity	NR		
	Autoimmune toxicity	NR		
	Treatment-related deaths	NR		
	Neuro toxicity	NR		
	Hematological toxicity	High-grade AEs mainly myelosuppression from CCRT; grade 3 anemia higher in TIL arm (7.7% vs 0)		
**Head and neck SCC**				
non-RCTs (Single-arm studies)				

NCT03083873[44]<br><br>USA	Grade ≥3 toxicity	Very common	NR	NR
	Cytokine-related toxicity	IL-2–related AEs frequent		
	Autoimmune toxicity	NR		
	Treatment-related deaths	NR		
	Neuro toxicity	Somnolence, syncope (low frequency)		
	Hematological toxicity	Febrile neutropenia, neutropenia, thrombocytopenia common across cohorts		
**Hepatocellular carcinoma**				
non-RCTs (Single-arm studies)				
Jiang SS[30]<br><br>China<br><br>2015	Grade ≥3 toxicity	No grade IV events	15/17 (88%)	~27.7 days (mean)
	Cytokine-related toxicity	No		
	Autoimmune toxicity	No		
	Treatment-related deaths	0		
	Neuro toxicity	NO		
	Hematological toxicity	Grade I–II leukopenia and neutropenia only		
**Osteosarcoma**				
non-RCTs (Single-arm studies)				
Wang C[45]<br><br>China<br><br>2022	Grade ≥3 toxicity	Yes	11/12 (92%)	NR
	Cytokine-related toxicity	Fever (10/11), IL-2 related		
	Autoimmune toxicity	NR		
	Treatment-related deaths	NR		
	Neuro toxicity	None observed		
	Hematological toxicity	Grade 4 lymphocytosis (8/11), neutropenia (2/11), thrombocytopenia (1/11)		

NR, not reported; IL-2, interleukin-2; TIL, tumor-infiltrating lymphocytes; CTCAE, Common Terminology Criteria for Adverse Events; DLT, dose-limiting toxicity; Cy, cyclophosphamide; Flu, fludarabine; TBI, total body irradiation

Supplementary Table 6: TIL therapy as a combination regimen: study characteristics and clinical outcomes

S. Table 6.1: Characteristics of selected studies: TIL therapy as a combinational option										
**• Author<br>• Country<br>• Year**	**• Study design<br>• Phase**	**• Intervention<br>• Control**	**• Participants enrolled<br>• Treated [IG/CG] n/n**	**• Cancer type<br>• Disease type**	**• Prior line of therapy<br>• Prior immunotherapy**	**Special population**	**• TIL Source<br>• TIL selection**	**Key regimen<br>• IL-2 Pre and post<br>• Cell dose*no. infusion**	**Lymphodepletion<br>• TBI**	**Outcome measured (primary and /or secondary outcomes)**
Combination of TIL with immune checkpoint inhibitors – Melanoma/ Metastatic melanoma										
Mullinax JE<br><br>USA<br><br>2018	Prospective single-arm interventional clinical trial<br><br>Early phase	Ipilimumab + lymphodepletion + TIL + IL-2<br><br>NR	13<br><br>12 (92%)	Metastatic melanoma<br>Unresectable stage III / IV melanoma,<br>M1a: 1<br>M1b: 2<br>M1c: 10	None for metastatic disease: 76.9%<br>Prior BRAF/MEK inhibitors: 2 patients<br>Prior ACT + anti-PD-L1 + chemotherapy: 1 patient<br>Checkpoint-naïve to ipilimumab (prior ACT allowed)	One ocular melanoma case	Surgically resected metastatic melanoma lesions<br><br>Fragment-derived bulk TIL<br><br>Tumor-reactive fragments identified by IFN-γ ELISA	High-dose IL-2 (720,000 IU/kg IV bolus q8–16 h; median 5 doses)<br>Median 6.5×10^{10}<br>Range 2.29×10^{10} – 1.04×10^{11} * 1	Cyclophosphamide 60 mg/kg/day × 2 days<br>Fludarabine 25 mg/m²/day × 5 days<br>No TBI	Primary: Safety and feasibility (≥60% receive TIL + ≥2 ipilimumab doses)<br><br>Secondary: NR
Hall MS<br><br>USA<br><br>2022	Prospective Phase I, two-cohort, single-arm trial	TIL ACT + nivolumab (with timing differences by cohort):<br><br>Cohort 1: Nivolumab after TIL infusion<br><br>Cohort 2: Nivolumab before tumor harvest,	11<br><br>9	Melanoma<br>Unresectable stage III / IV metastatic melanoma	Majority treatment-naïve, some prior adjuvant IFN, ipilimumab, or BRAF/MEK therapy<br>No prior anti-PD-1 allowed	Anti-PD-1–naïve metastatic melanoma	Surgically resected metastatic melanoma lesions<br><br>Fragment culture<br>Tumor reactivity assessed by IFN-γ release	High-dose IL-2<br><br>Mean: 8.5×10^{10} cells<br>Range: 5.3×10^{10} – 1.3×10^{11} * 1	Cyclophosphamide 60 mg/kg/day ×2 (day −7, −6)<br>Fludarabine 25 mg/m²/day ×5 (day −5 to −1)<br>No	Primary: Safety and feasibility (DLT-based)<br><br>Secondary: Objective response rate, Progression-free survival, Overall survival, TIL persistence and clonality

		during TIL generation, and after TIL<br>NR								
L'Orphelin JM<br><br>France<br><br>2024	Prospective, exploratory, open-label, non-randomised, uncontrolled clinical trial Phase I	Neoadjuvant nivolumab → TIL infusion(s) + IL-2 → adjuvant nivolumab<br><br>NR	10<br>Sample size (Treated – any therapy): 7 (received nivolumab), Sample size (Treated – TIL + nivolumab): 4	Melanoma Advanced/metastatic melanoma (AJCC IIIb–IV)	None (treatment-naïve population)<br><br>NR	Untreated metastatic melanoma; included acral, mucosal, NRAS- and BRAF-mutant subtypes	Resected metastatic lymph nodes (single macroscopic LN)<br>Bulk TILs; no antigen-specific preselection	IL-2 (Proleukin®) 6 MIU/day for 5 days after each TIL infusion<br>0.5×10^9 – 20×10^9 TILs per injection *<br>Planned: 2<br>Actual: 1–2 (one patient received only one due to low yield)	No lymphodepletion<br><br>No TBI	Primary: Safety and biological tolerability of TIL + anti-PD-1 therapy<br><br>Secondary: Objective response, PFS, OS, duration of response
Hasanov M<br><br>USA<br><br>2025	Randomized Phase II clinical trial	Arm-1: TIL + high-dose IL-2 + pembrolizumab (n = 7)<br><br>Arm-2: TIL + low-dose IL-2 + pembrolizumab (n = 7)	14<br><br>14	Metastatic melanoma (cutaneous, mucosal, uveal, unknown primary)- Stage IV (M1c–M1d predominance)	Median 3 prior systemic therapies (range 1–6)<br><br>79% prior checkpoint inhibitors (anti-PD-1 ± anti-CTLA-4)	Heavily pretreated, mostly ICI-refractory melanoma	Surgically resected metastatic melanoma lesions<br><br>Bulk TIL (no enrichment or tumor-reactivity selection for infusion)	Arm-1: High-dose IL-2 (720,000 IU/kg IV q8–16h, ≤15 doses)<br>Arm-2: Low-dose IL-2 (2 million IU SC daily × 14 days)<br><br>Median 17.3×10^9 cells (range 1.5–93.17) * 1	Cyclophosphamide 60 mg/kg/day × 2<br>Fludarabine 25 mg/m²/day × 5<br><br>No TBI	Primary: Objective response rate (ORR) per RECIST 1.1<br><br>Secondary: NR
Combination of TIL with immune checkpoint inhibitors – Metastatic Breast										
Zacharakis N<br>USA<br>2022	Prospective single-arm trial	Selected neoantigen-reactive TIL + lymphodepletion + IL-2 + pembrolizumab	6<br><br>6	Metastatic breast cancer (mBrCa)<br>Treatment-refractory metastatic disease (metastatic epithelial cancer	Median 4 prior lines (range 1–10) in the screened cohort<br>NR	BRCA germline status discussed at cohort level (BRCA+ subgroup present), but treated-	Resected metastatic lesion(s); TIL grown from tumor fragments (sites included liver/lung/LN/soft tissue at	Aldesleukin 720,000 IU/kg every 8 hours to tolerance<br><br>Total infused cells ranged 3.06×10^{10} to 1.04×10^{11} (median	Cyclophosphamide 60 mg/kg daily on days –7 and –6, Fludarabine 25 mg/m² daily from day –7 to	Primary: Objective response rate (ORR)<br><br>Secondary: Exploratory endpoints for tissue harvest

				protocol cohort; breast cancer cohort reported)		subset breakdown NR	cohort level)	6.00×10^{10}) * 1 infusion	day –3<br><br>No TBI	protocol: successful TIL growth and identification of neoantigen-reactive TIL (assay-based)
Combination of TIL with immune checkpoint inhibitors – Gastrointestinal carcinoma										
Lowery, F.J<br><br>USA<br><br>2025	Non-randomized, single-arm Phase II trial with sequential cohorts	Adoptive TIL transfer ± pembrolizumab<br><br>NR	Total treated 91<br>Bulk unselected TIL 18<br>Selected TIL (SEL-TIL) 39<br>Selected TIL + Pembrolizumab (SEL-TIL + P) 34<br>Total treated 91	Gastrointestinal epithelial cancers Metastatic, treatment-refractory, MMR-proficient GI cancers. Colorectal (majority) Gastric, esophageal, pancreatic, and cholangiocarcinoma	Median 4–5 prior systemic regimens<br>Rare (0–12%)	Early-onset colorectal cancer common; MSI-H excluded	Resected metastatic tumor tissue<br><br>Bulk arm: Unselected TILs<br>SEL-TIL arms: Neoantigen-reactive fragment-selected TILs	High-dose IL-2 (720,000 IU/kg q8–12 h)<br>SEL-TIL: median 7.3×10^{10}<br>SEL-TIL + P: median 8.5×10^{10} * 1	Cyclophosphamide 60 mg/kg × 2 days<br>Fludarabine 25 mg/m^2 × 5 days<br><br>No TBI	Primary: Objective response rate (RECIST 1.0)<br><br>Secondary: Toxicity
Combination of TIL with Dendritic vaccine – Metastatic melanoma										
Saberian C<br><br>USA<br><br>2021	Randomized open-label phase II trial	Lymphodepletion + TIL + MART-1–pulsed dendritic cell (DC) vaccine + high-dose IL-2<br><br>Lymphodepletion + TIL + high-dose IL-2	12/11<br><br>10/8	Melanoma Metastatic melanoma (Stage III/IV; majority M1c)	≥1 prior systemic therapy: 89%; Treatment-naïve: 11%<br>1 patient previously treated with ipilimumab	HLA-A*0201–positive, MART-1–reactive TIL required, Included patients with stable brain metastases	Resected metastatic melanoma tumor deposits ≥0.1% MART-1–reactive $CD8^{+}$ T cells	High-dose IL-2 (720,000 IU/kg IV q8h)<br><br>Median 60.7 billion (range 1.7–130), Higher in TIL+DC arm * Single dose	Cyclophosphamide 60 mg/kg/day ×2, Fludarabine 25 mg/m^2/day ×5<br><br>No TBI	Primary: Persistence of MART-1–specific TIL+ dc vaccine IL2<br>Secondary: Objective response, OS, and PFS
Combination of TIL with recombinant interferon alfa										

Queirolo P<br><br>Italy<br><br>1999	Pilot interventional study	Autologous TIL + rIL-2 + rIFN-α2a	36<br><br>19	Melanoma Stage IV unresectable/metastatic	Chemotherapy 42%; limb perfusion 15%; biotherapy 24%; treatment-naïve 15%<br>Yes (biotherapy in 24%)	Outpatient-treated metastatic melanoma	Metastatic lymph nodes (15), subcutaneous metastases (4)<br>Bulk polyclonal TIL	rIL-2 (SC, intermediate dose)<br><br>Median 18 × 10^9 (range 1–43 × 10^9) * Single infusion	No lymphodepletion<br><br>No TBI	Primary: Activity, feasibility, and toxicity<br>Secondary: NR

S. Table 6.2: Primary outcomes of included studies- TIL therapy as a combinational option

Combination of TIL with immune checkpoint inhibitors – Melanoma/ Metastatic melanoma

• Author<br>• Country<br>• Year	Clinical efficiency outcomes		Outcome observed	Response criteria	Medium Follow-up	Duration of response	Time to respond
Hall MS<br><br>USA<br><br>2022	Objective response rate		(ORR): 36% (4/11, ITT)	RECIST 1.1	NR	All responders >18 months<br>Two responses ongoing at 46+ and 71+ months	NR
	Complete response rate		0				
	Partial response rate		0.36				
	Disease control rate		NR				
	Progression-free survival	Months	Cohort 1: 5 months<br>Cohort 2: 12.5 months				
		HR	NR				
	Overall survival	Months	Median OS: 23 months				
		HR	NR				
Mullinax JE<br><br>USA<br><br>2018	Objective response rate		38% (5/13) by intention-to-treat at 12 weeks	RECIST v1.1	35 months	4 responses ongoing >34–52+ months	NR
	Complete response rate		1/13 (7.7%) (converted from PR at 52 months)				
	Partial response rate		4/13 (30.8%)				
	Disease control rate		8/13 (61.5%)				
	Progression-free survival	Months	7.3 months (95% CI 6.1–29.9)				

		HR	NR				
	Overall survival	Months	NR				
		HR	NR				
Hasanov M<br><br>USA<br><br>2025	Objective response rate		Overall: 14%<br>Arm-1: 7%<br>Arm-2: 7%	RECIST 1.1	9.2 months	One PR ongoing >76 months (Arm-2)	NR
	Complete response rate		0				
	Partial response rate		2/14 (14%)				
	Disease control rate		Overall: 29%				
	Progression-free survival	Months	Arm-1: 3.9 months<br>Arm-2: 2.1 months<br>Overall: 2.8 months				
		HR	NR				
	Overall survival	Months	Arm-1: 9.7 months<br>Arm-2: 8.8 months<br>Overall: 9.2 months				
		HR	NR				
L'Orphelin JM<br><br>France<br><br>2024	Objective response rate		75% (3/4)	RECIST	560 days (range 274–1,240)	9 months – 3.4 years (ongoing CRs)	NR
	Complete response rate		2/4 (50%) (CR at end of study or during follow-up)				
	Partial response rate		1/4 (25%)				
	Disease control rate		NR (SD not pooled explicitly)				
	Progression-free survival	Months	Mean PFS: 615 days				
		HR	NR				
	Overall survival	Months	NR				
		HR	NR				
Combination of TIL with immune checkpoint inhibitors – Metastatic Breast							
Zacharakis N<br>USA<br>2022	Objective response rate		3/6 objective tumor regression (50%)	RECIST 1.0	NR	CR: 66+ months (ongoing), PR: 6 months and 10 months	NR
	Complete response rate		1/6 (CR ongoing 66+ months)				
	Partial response rate		2/6 (PR 10 months)				
	Disease control rate		NR				
	Progression-free survival	Months	NR				
		HR	NR				
		Months	NR				

	Overall survival	HR	NR				
Combination of TIL with immune checkpoint inhibitors – Gastrointestinal carcinoma							
Lowery, F.J<br><br>USA<br><br>2025	Objective response rate		Bulk TIL 0%<br>SEL-TIL 7.7%<br>SEL-TIL + P 23.5%	RECIST 1.0	NR	SEL-TIL: 8–70+ months<br>SEL-TIL + P: 4–42+ months	NR
	Complete response rate		Bulk TIL 0%<br>SEL-TIL 0%<br>SEL-TIL + P 0%				
	Partial response rate		Bulk TIL 0%<br>SEL-TIL 3/39<br>SEL-TIL + P 8/34				
	Disease control rate		NR				
	Progression-free survival	Months	NR				
		HR	NR				
	Overall survival	Months	NR				
		HR	NR				
Combination of TIL with Dendritic vaccine – Metastatic melanoma							
Saberian C<br><br>USA<br><br>2021	Objective response rate		Overall: 39%,<br>TIL: 30% (3/10),<br>TIL+DC: 50% (4/8)	irRC	2.2 years	Median ~0.8 years	NR
	Complete response rate		TIL: 10%,<br>TIL+DC: 12.5%				
	Partial response rate		TIL: 20%,<br>TIL+DC: 37.5%				
	Disease control rate		TIL: 70%,<br>TIL+DC: 75%				
	Progression-free survival	Months	TIL: 0.26 years,<br>TIL+DC: 0.49 years				
		HR	NR				
	Overall survival	Months	TIL: 4.1 years,<br>TIL+DC: 2.0 years				
		HR	NR				
Combination of TIL with recombinant interferon alfa							
Queirolo P<br><br>Italy	Objective response rate		11% (2/19)	WHO criteria	NR	CRs: 8 and 30 months	NR
	Complete response rate		11% (2/19)				
	Partial response rate		0				
	Disease control rate		63% (CR + SD)				

1999	Progression-free survival	Months	NR				
		HR	NR				
	Overall survival	Months	NR				
		HR	NR				

S. Table 3.3: Secondary outcomes of included studies					
• Author<br>• Country<br>• Year	Safety outcomes	Outcome observed	Manufacturing success rate	Vein to vein time
Combination of TIL with immune checkpoint inhibitors – Melanoma/ Metastatic melanoma				
Hall MS<br><br>USA<br><br>2022	Grade ≥3 toxicity	Yes	100% (no TIL failures)	NR
	Cytokine-related toxicity	IL-2–related toxicities common		
	Autoimmune toxicity	Grade 3 colitis (nivolumab-related, outside DLT window)<br>Grade 3 rash		
	Treatment-related deaths	0		
	Neuro toxicity	NR		
	Hematological toxicity	Universal transient cytopenias from lymphodepletion		
Mullinax JE<br><br>USA<br><br>2018	Grade ≥3 toxicity	Yes	100% TIL growth from resected tumors	Mean 51 days (range 39–77)
	Cytokine-related toxicity	IL-2-related hypotension, hypoxemia, electrolyte disturbances		
	Autoimmune toxicity	Hypothyroidism: 3, Hepatitis: 2, Uveitis: 1, Colitis: 1		
	Treatment-related deaths	0		
	Neuro toxicity	Presyncope/syncope (Grade 3): 1		
	Hematological toxicity	Febrile neutropenia: 10/12, Leukopenia, thrombocytopenia: common		
Hasanov M<br><br>USA<br><br>2025	Grade ≥3 toxicity	Yes	NR	NR
	Cytokine-related toxicity	Febrile neutropenia: 57% both arms		
	Autoimmune toxicity	Hypothyroidism, vitiligo (low frequency)		

	Treatment-related deaths	0		
	Neuro toxicity	Confusion, dizziness, hallucinations (Grade 1–2)		
	Hematological toxicity	Universal cytopenias post-lymphodepletion		
L'Orphelin JM<br><br>France<br><br>2024	Grade ≥3 toxicity	1 grade-3 AE related to treatment (myocardial infarction attributed to nivolumab)	4/7 treated patients successfully received TILs	NR
	Cytokine-related toxicity	Mild IL-2-related events; no CRS reported		
	Autoimmune toxicity	Vitiligo, endocrine AEs (low-grade)		
	Treatment-related deaths	0		
	Neuro toxicity	Confusion, hallucinations (low-grade)		
	Hematological toxicity	Minimal (no lymphodepletion)		
Combination of TIL with immune checkpoint inhibitors – Gastrointestinal carcinoma				
Lowery, F.J<br><br>USA<br><br>2025	Grade ≥3 toxicity	1	95/168 screened (57%) yielded reactive TILs	Median screening time 2.6 months
	Cytokine-related toxicity	IL-2–related expected events		
	Autoimmune toxicity	Pembrolizumab-related colitis, pneumonitis (rare)		
	Treatment-related deaths	1 (1.1%) – adenoviral hepatitis		
	Neuro toxicity	ICU-level complications in 10%		
	Hematological toxicity	Universal grade ≥ 3 cytopenias		
Combination of TIL with Dendritic vaccine – Metastatic melanoma				
Saberian C<br><br>USA<br><br>2021	Grade ≥3 toxicity	Expected chemo + IL-2 toxicities	8/23 treated successfully (≈78%)	NR
	Cytokine-related toxicity	IL-2–related, reversible		
	Autoimmune toxicity	Vitiligo (1 CR patient, TIL+DC)		
	Treatment-related deaths	NR		
	Neuro toxicity	NR		
	Hematological toxicity	NR		
Combination of TIL with recombinant interferon alfa				
Queirolo P<br><br>Italy	Grade ≥3 toxicity	Yes	50% (19/38 samples)	Median 52 days
	Cytokine-related toxicity	Yes (IL-2 / IFN-α related)		
	Autoimmune toxicity	NR		
	Treatment-related deaths	NO		

1999	Neuro toxicity	NO		
	Hematological toxicity	Anemia, neutropenia, thrombocytopenia (each 11%)		
