# Supplementary material for: Efficacy, safety, and characteristics of adoptive tumor-infiltrating lymphocyte therapy in solid tumours: a systematic review and meta-analysis
Source: Front Oncol. 2026 Jul 22;16:1859306. doi: 10.3389/fonc.2026.1859306 (PMC13437784; doi:10.3389/fonc.2026.1859306)
Supplement: Supplementary file 2 [file DataSheet1.docx]

**Appendix A: Details on search strategies applied in various databases**

| **Database** | **Search details** | **Results** |
| --- | --- | --- |
| **PubMed®** | (("Neoplasms"[Mesh] OR cancer* OR tumor* OR tumour* OR neoplasm* OR carcinoma* OR malignan*) AND ("Lymphocytes, Tumor-Infiltrating"[Mesh] OR "tumor infiltrating lymphocyte*" OR "tumour infiltrating lymphocyte*" OR TIL OR TILs OR "adoptive cell therap*" OR "adoptive immunotherap*" OR "cellular immunotherap*"))  Filters: Humans Date: Inception to 31 December 2025 | 122 |
| **Scopus®** | (TITLE-ABS-KEY (cancer OR tumor OR tumour OR neoplasm OR carcinoma OR malignancy)) AND (TITLE-ABS-KEY ("tumor infiltrating lymphocyte*" OR "tumour infiltrating lymphocyte*" OR TIL OR TILs OR "adoptive cell therap*" OR "adoptive immunotherap*" OR "cellular immunotherap*")) | 451 |
| **Cochrane Library Central®** | #1 MeSH descriptor: [Neoplasms] explode all trees  #2 (cancer OR tumor OR tumour OR neoplasm OR carcinoma OR malignancy):ti,ab,kw  #3 MeSH descriptor: [Lymphocytes, Tumor-Infiltrating] explode all trees  #4 ("tumor infiltrating lymphocyte*" OR "tumour infiltrating lymphocyte*" OR TIL OR TILs):ti,ab,kw  #5 ("adoptive cell therap*" OR "adoptive immunotherap*" OR "cellular immunotherap*"):ti,ab,kw  #6 #3 OR #4 OR #5  #7 #1 OR #2  #8 #6 AND #7  **Filters applied:**  Search field: Title Abstract Keyword  Document type: Trials (Word variations have been searched) | 18 |
| **WHO ICTRP** | Above mentioned search terms were adopted and suitably applied to amend necessary documents | 244 |
| **Other potential sources** | Above mentioned search terms were adopted and suitably applied to amend necessary documents | 5 |

**Appendix B: Details of PICOS format for study inclusion criteria**

| **PICOS format** | |
| --- | --- |
| **Participants** | - Adult patients with solid tumors (any histological type and disease stage) receiving adoptive cell therapy using autologous tumor-infiltrating lymphocytes (TILs). |
| **Intervention** | - Adoptive TIL therapy involves tumor-derived tumor-infiltrating lymphocytes isolation, ex vivo expansion (with or without selection or enrichment), and reinfusion into the patient, typically following lymphodepleting chemotherapy with or without interleukin-2 (IL-2) support and/or immune checkpoint inhibitors. |
| **Comparators** | - Any comparator or no comparator, including standard of care, other systemic therapies, or single-arm studies without a control group. |
| **Outcomes** | Primary outcomes:   - Objective response rate (ORR), including complete response (CR) and partial response (PR) - Progression-free survival (PFS) and overall survival (OS), where available   Secondary outcomes:   - Treatment-related adverse events (particularly grade ≥3 toxicity) - Treatment-related mortality - Manufacturing and treatment feasibility metrics, including manufacturing success rate and vein-to-vein time |
| **Study designs** | - Randomized controlled trials, non-randomized clinical trials, prospective cohort studies, and retrospective cohort studies evaluating TIL therapy in solid tumors. |

**Appendix C: Details of excluded literatures**

| **S.No** | **First author/registry / year** | **Title of the study** | **Reason for exclusion** |
| --- | --- | --- | --- |
| 1 | NCT05576077 | A Study of TBio-4101 (TIL) and Pembrolizumab in Patients with Advanced Solid Tumors | Terminated trial |
| 2 | NCT03992326 | Adoptive Transfer of Autologous Tumor-Infiltrating Lymphocytes in Solid Tumors | Terminated trial |
| 3 | NCT02111863 | Immunotherapy Using 41BB Selected Tumor Infiltrating Lymphocytes for Patients With Metastatic Melanoma | Terminated trial |
| 4 | NCT01369875 | Modified Tumor Infiltrating Lymphocytes for Metastatic Melanoma | Terminated trial |
| 5 | NCT02375984 | A Study Using Tumor-Reactive Autologous Tumor Infiltrating Lymphocytes (TIL) in Metastatic Melanomas | Terminated trial |
| 6 | NCT01236573 | Cell Therapy for Metastatic Melanoma Using CD8 Enriched Tumor Infiltrating Lymphocytes | Terminated trial |
| 7 | NCT00863330 | Laboratory-Treated Autologous Lymphocytes and Aldesleukin After Cyclophosphamide and Fludarabine in Treating Patients With Metastatic Melanoma | Terminated trial |
| 8 | NCT00509496 | Phase II Study of Metastatic Melanoma With Lymphodepleting Conditioning and Anti-gp100:154-162 TCR Gene Engineered Lymphocytes | Terminated trial |
| 9 | NCT06190249 | Efficacy & Safety for LN144 With Pembrolizumab With High Risk Stage IIIb-d Resectable Melanoma | Terminated trial |
| 10 | Peyret C; France; 1991 | Adoptive immunotherapy and metastasized cancer of the kidney. Regulating effects of interleukin-4 on tumor-infiltrating lymphocytes (TIL) | Article in French; old IL-4–based protocol; non-comparable to contemporary TIL therapy |
| 11 | Semino C; Italy; 1999 | Adoptive immunotherapy of advanced solid tumors: An eight year clinical experience | Does not meet PICOS; mixed advanced solid tumors and non-standard historical protocol |
| 12 | Horváth J; 1994 | Autologous activated lymphocyte therapy in a community hospital | Does not meet PICOS; activated lymphocyte therapy rather than defined TIL ACT |
| 13 | EUCTR2007-000776-16-CZ; 2014 | Development of Tumor Infiltrating Lymphocytes in Patients with Metastatic Melanoma - TIL | No results published / no registry results available |
| 14 | Aebersold P; USA; 1991 | Lysis of autologous melanoma cells by tumor-infiltrating lymphocytes: association with clinical response | Mechanistic/correlative study; not a standardized clinical efficacy trial |
| 15 | Besser MJ; Israel; 2009 | Minimally cultured or selected autologous tumor-infiltrating lymphocytes after a lympho-depleting chemotherapy regimen in metastatic melanoma patients | Duplicate / overlapping melanoma TIL cohort relative to later reports |
| 16 | Bayley EM; USA; 2021 | Pulmonary resection for tissue harvest in adoptive tumor-infiltrating lymphocyte therapy: Safety and feasibility | Surgical feasibility study; does not report therapeutic efficacy outcomes |
| 17 | NL-OMON34085; Netherlands; 2010 | Randomized phase II study using a non-myeloablative lymphocyte depleting regimen of chemotherapy followed by infusion of tumor infiltrating lymphocytes and interleukin-2 in metastatic melanoma | Registry trial with insufficient publicly extractable results / no full results publication identified |
| 18 | NCT06566092 | Study of Autologous Tumor-Infiltrating Lymphocytes in Pediatric, Adolescent, and Young Adult Participants | Ongoing / recruiting trial with no mature results available |
| 19 | Tas L; 2025 | Surgical considerations for tumour-infiltrating lymphocyte therapy in melanoma: results from a randomized phase III trial | Surgical/technical focus; not an efficacy report of TIL treatment outcomes |
| 20 | NCT03158935 | The ACTIVATE Trial | Early-phase combination trial with heterogeneous cohorts and insufficient mature data for inclusion |
| 21 | IRCT20140818018842N34; Iran; 2023 | Investigating the safety and efficacy of cell therapy with Tumor-Infiltrating Lymphocytes (TILs) in Triple Negative Breast Cancer | Pilot single-arm study with very small sample size and no mature published full article |
| 22 | Dudley ME; USA; 2001 | Adoptive transfer of cloned melanoma-reactive T lymphocytes for the treatment of patients with metastatic melanoma | Does not meet PICOS; cloned T-cell therapy rather than bulk autologous TIL therapy |
| 23 | Figlin RA; 1992 | Cancer immunotherapy using tumor-infiltrating lymphocytes | Conference paper / non-full article primary report |
| 24 | CTRI/2025/09/095486 | Correlation of Tumor Infiltrating Lymphocytes and immune markers with pathological features in Oral Squamous Cell Carcinoma | Observational biomarker study; no therapeutic TIL intervention |
| 25 | Donia M; 2013 | Methods to improve adoptive T-cell therapy for melanoma: IFN-γ enhances anticancer responses of cell products for infusion | Translational/manufacturing-focused study; not a clinical efficacy study |
| 26 | Galore-Haskel G; 2021 | microRNA expression patterns in tumor infiltrating lymphocytes are strongly associated with response to adoptive cell transfer therapy | Biomarker/correlative study; not eligible clinical efficacy report |
| 27 | NCT05941936; China; 2023 | A Clinical Study on LM103 for the Treatment of Advanced Solid Tumors | Recruiting early-phase trial with no mature results available |
| 28 | EUCTR2018-001608-12-GB | A Phase 2, Multicenter Study of Autologous Tumor Infiltrating Lymphocytes (LN-144 or LN-145) in Patients with Solid Tumors | No publicly available full results suitable for extraction |
| 29 | ChiCTR2200058937; China; 2022 | Gene modified tumor infiltrating lymphocyte in gynecological malignancies | Does not meet PICOS; genetically modified TIL product, not conventional autologous TIL therapy |
| 30 | NL-OMON52190 | A Phase 2 Multicenter Study of Autologous Tumor Infiltrating Lymphocytes (LN-145) in Patients with Metastatic NSCLC | No mature published results / insufficient extractable data |
| 31 | NCT02621021 | A Phase 2 Trial for Metastatic Melanoma Using Adoptive Cell Therapy With Tumor Infiltrating Lymphocytes Plus IL-2 Either Alone or Following the Administration of Pembrolizumab | Recruiting / ongoing; no final results available |
| 32 | NCT04383067 | A Phase 2, Single-Center, Open Label Study… in Metastatic Urothelial Carcinoma Patients | Recruiting / ongoing; no mature results available |
| 33 | Jiang SS; China; 2015 | A phase I clinical trial utilizing autologous tumor-infiltrating lymphocytes in patients with primary hepatocellular carcinoma | Small phase I study with limited sample size and insufficient data for pooled synthesis |
| 34 | NCT05417750 | A Phase I Study of GC101 TIL in Advanced Solid Tumors | Phase I study; immature data and highly heterogeneous tumor population |
| 35 | CTIS2024-512877-28-00 | A Phase I Study Using Hepatic Arterial Infusion of Autologous Tumor Infiltrating Lymphocytes in Patients with Melanoma and Liver Metastases | Ongoing early-phase trial with no available results; non-standard delivery route |
| 36 | Ravaud A; France; 1995 | A phase I trial of repeated tumour-infiltrating lymphocyte (TIL) infusion in metastatic melanoma | Historical phase I study with outdated, non-standardized protocol and non-comparable outcomes |
| 37 | NCT04426669 | TIL for metastatic gastrointestinal cancers with CISH inhibited using CRISPR | Does not meet PICOS; gene-edited TIL product rather than conventional TIL therapy |
| 38 | NCT03935893 | Adoptive Transfer of Tumor Infiltrating Lymphocytes for Advanced Solid Cancers | Recruiting / ongoing; no mature results available |
| 39 | NCT03419559 | Study of Autologous Tumor Infiltrating Lymphocytes (LN-145) In Combo With Durvalumab in NSCLC | Combination early-phase/phase II study with no mature published extractable results |
| 40 | NCT05098184 | A Study of GC101 TIL in Advanced Melanoma | Recruiting trial with no final outcome data available |
| 41 | CTIS2023-506400-99-00 | The PragmaTIL | Ongoing protocol/trial registration only; no results available |
| 42 | Martin-Liberal J; 2024 | Clinical protocol phase II study of tumor infiltrating lymphocytes in advanced tumors with alterations in the SWI/SNF complex: the TILTS study | Protocol paper; no patient results reported |
| 43 | NCT06961357 | Clinical Trial of CD40L-augmented TIL for Patients with Advanced Melanoma | Recruiting trial; genetically augmented TIL product and no mature results available |
| 44 | ScTIL-B-009-2022 | ScTIL injection in digestive system malignant solid tumors | Does not meet PICOS; genetically modified / lentiviral ScTIL product rather than standard autologous TIL therapy |
| 45 | IOV-MEL-202 / CTIS2025-522054-40-00 | Lifileucel in melanoma | Registry-only / no mature publicly extractable full results in the screened source set |
| 46 | STIL101 / NCI-2024-08121 | Therapeutic tumor infiltrating lymphocytes in advanced solid tumors | Protocol/ongoing study with no mature clinical results available |
| 47 | OVACURE | Ovarian cancer TIL study | No mature published full results available in screened records |
| 48 | NCT02500576 | TURNSTILE II/pembrolizumab plus TIL in melanoma | Duplicate/updated record; linked trial results record rather than distinct full-text study for separate inclusion |
| 49 | Mullinax JE; USA  2018 | Combination of TIL with immune checkpoint inhibitors – Melanoma/ Metastatic melanoma | Does not meet PICOS. |
| 50 | Future Oncol protocol paper; 2024 | TILTS study | Protocol article only; no clinical results available |
| 51 | CD40L-augmented TIL protocol record | Phase I/II Clinical Trial of CD40L-augmented Tumor Infiltrating Lymphocytes for Advanced Melanoma | Ongoing / recruiting with no mature results available |
| 52 | Hepatic arterial infusion TIL protocol | A Phase I Study Using Hepatic Arterial Infusion of Autologous TIL in Melanoma Liver Metastases | Trial registration only; no published results and non-standard regional infusion approach |
| 53 | Goff SL; USA; 2016 | A phase I clinical trial utilizing autologous tumor-infiltrating lymphocytes in patients with primary hepatocellular carcinoma | Total body irradiation was the intervention being compared with non-myeloablative chemotherapy |
| 54 | Dudley ME; 2010 | CD8+ enriched "young" tumor infiltrating lymphocytes can mediate regression of metastatic melanoma. | Total body irradiation was the intervention being compared with non-myeloablative chemotherapy alone |
| 55 | JPRN-UMIN000011431 | A feasibility study using short-term cultured anti-tumor autologous lymphocytes in metastatic melanoma | Registry record with no published full results |
| 56 | NCT06598371 | KSQ-004EX engineered TIL trial in advanced solid tumors | Genetically modified TIL product; does not meet PICOS |
| 57 | Dudley ME, 2002 | Nonmyeloablative chemotherapy + adoptive T cell transfer in melanoma | Patient duplication |
| 58 | NCT01462903 | A Study of Adoptive Immunotherapy With Autologous Tumor Infiltrating Lymphocytes in Solid Tumors | Recruiting / registry-only record with no mature published full results |
| 59 | NCT00338377 / MAT02 | Randomized phase II trial of lymphodepletion plus adoptive cell transfer of tumor-infiltrating lymphocytes, with or without dendritic cell vaccination, in patients with metastatic melanoma | Registry-only / insufficient publicly extractable results |
| 60 | EUCTR2016-003447-11-GB | A Phase 2, Multicenter Study to Evaluate the Efficacy and Safety Using Autologous Tumor Infiltrating Lymphocytes (LN-145) in Patients with recurrent, metastatic or persistent cervical carcinoma | Registry record with insufficient full publicly extractable results for review inclusion. |
| 61 | Verdecchia GM; 2003 | Adjuvant adoptive immunotherapy in patients with stage III and resected stage IV melanoma: a pilot study | Extractable outcome data not available. |
| 62 | NCT04217473 / TUNINTIL | TNFalpha and Interleukin 2 Coding Oncolytic Adenovirus TILT-123 During TIL Treatment of Advanced Melanoma | Combination oncolytic-virus plus TIL early-phase study; no mature extractable outcomes. |
| 63 | ChiCTR2000030353 | Treatment of advanced liver cancer with tumor infiltrating lymphocytes by transcatheter arterial infusion | Ongoing registry study; non-standard arterial infusion approach and no mature full results |
| 64 | KCT0010263 | Safety evaluation of autologous tumor-infiltrating lymphocytes (TILs) therapy for recurrent ovarian cancer | Recruiting registry study with no mature published results |
| 65 | ChiCTR2100044420 | Clinical study on the safety and efficacy of E/S(c)TIL (Gene-modified tumor infiltrating lymphocytes) in the treatment of malignant solid tumors | Gene-modified TIL product; does not meet PICOS. |
| 66 | Fujian Cancer Hospital; China; 2021 | TIL therapy in melanoma and hepatocellular carcinoma | No mature results |
| 67 | TILT-T215; Finland; 2020 | TILT-123 protocol record | No mature results |
| 68 | MM-TIL-P1/2; Japan; 2017 | Adoptive TIL therapy with lymphodepletion in melanoma | Registry-only; no full results available |
| 69 | KSQ-004EX (duplicate); USA; 2024 | KSQ-004EX TIL therapy | Duplicate registry record |
| 70 | NR; USA | Cell-based therapy for skin cancer | Duplicate registry description |
| 71 | NR; USA | Randomized trial using autologous lymphocytes in skin cancer | Duplicate registry description |
| 72 | NR; China; 2021 | E/S(c)TIL study description | Duplicate registry entry |
| 73 | NR; South Korea; 2021 | Ovarian cancer TIL registry description | Duplicate registry entry |
| 74 | NR; China; 2020 | Liver cancer arterial TIL study description | Duplicate registry entry |
| 75 | Mushtaq MU; USA; 2025 | Reduced-dose lymphodepletion before TIL therapy | Methodological study; overlapping cohort |
| 76 | NR; USA; 2022 | Neoantigen-specific TIL trial in GI cancers | Not conventional TIL therapy (neoantigen-selected) |
| 77 | NR; USA | Mesothelioma TIL trial | Early-phase registry; no extractable results |
| 78 | NCT03158935; USA; 2017 | ACTIVATE trial | Early-phase heterogeneous study; insufficient data |
| 79 | Hall MS; USA  2022 | Combination of TIL with immune checkpoint inhibitors – Melanoma/ Metastatic melanoma | Does not meet PICOS. |
| 81 | Donia M; Denmark; 2013 | TIL manufacturing optimization study | Translational study; no clinical efficacy outcomes |
| 82 | Galore-Haskel G; Israel; 2021 | miRNA in TIL therapy response | Biomarker/correlative study |
| 83 | Martin-Liberal J; UK; 2024 | TILTS study protocol | Protocol paper; no patient results |
| 84 | CTIS2023-506400-99-00; Europe; 2023 | PragmaTIL trial | Ongoing trial; no results available |
| 85 | STIL101 / NCI-2024-08121; USA; 2024 | TIL therapy in advanced solid tumors | Ongoing study; no results |
| 86 | OVACURE; Europe | Ovarian cancer TIL study | No mature published results |
| 87 | NCT05098184; China; 2022 | GC101 TIL in melanoma | Recruiting; no outcome data |
| 88 | NCT03935893; USA; 2019 | TIL therapy in advanced solid cancers | Ongoing; no extractable results |
| 89 | Queirolo P Italy  1999 | Combination of TIL with recombinant interferon alfa | Does not meet PICOS. |
| 90 | Saberian C; USA  2021 | Combination of TIL with Dendritic vaccine – Metastatic melanoma | Does not meet PICOS. |
| 91 | Lowery, F.J; USA  2025 | Combination of TIL with immune checkpoint inhibitors – Gastrointestinal carcinoma | Does not meet PICOS. |
| 92 | Hasanov M; USA  2025 | Combination of TIL with immune checkpoint inhibitors – Melanoma/ Metastatic melanoma | Does not meet PICOS. |
